# Translational incorporation of modified phenylalanines and tyrosines during cell-free protein synthesis†

**DOI:** 10.1039/d0ra00655f

**Published:** 2020-03-18

**Authors:** Zhongqiang Wang, Hayden Matthews

**Affiliations:** Key Laboratory of Biocatalysis & Chiral Drug Synthesis of Guizhou Province, Generic Drug Research Center of Guizhou Province, School of Pharmacy, Zunyi Medical University Zunyi 563000 China zqwang@zmu.edu.cn; Key Laboratory of Basic Pharmacology of Ministry of Education and Joint International Research Laboratory of Ethnomedicine of Ministry of Education, Zunyi Medical University Zunyi 563000 China; Research School of Chemistry, Australian National University 137 Sullivans Creek Road Acton ACT 2601 Australia; Australian National University Medical School 54 Mills Road Acton ACT 2601 Australia Hayden.Matthews@anu.edu.au

## Abstract

Inherent promiscuity of bacterial translation is demonstrated by mass spectrometric quantification of the translational incorporation of ring-substituted phenylalanines and tyrosines bearing fluoro-, hydroxyl-, methyl-, chloro- and nitro-groups in an *E. coli*-derived cell-free system. Competitive studies using the cell-free system show that the aminoacyl-tRNA synthetases (aaRS) have at least two orders of magnitude higher specificity for the native substrate over these structural analogues, which correlates with studies on the purified synthetase.

## Abstract 2

*E. coli* wild-type translational machinery utilizes a range of nonproteinogenic amino acids for protein synthesis with incorporation levels greater than 95%.

## Graphical abstract

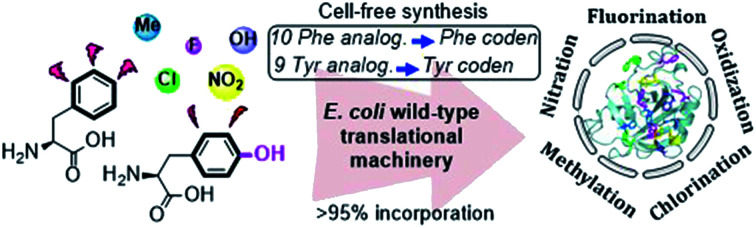

## Introduction

**Fig. 1:**
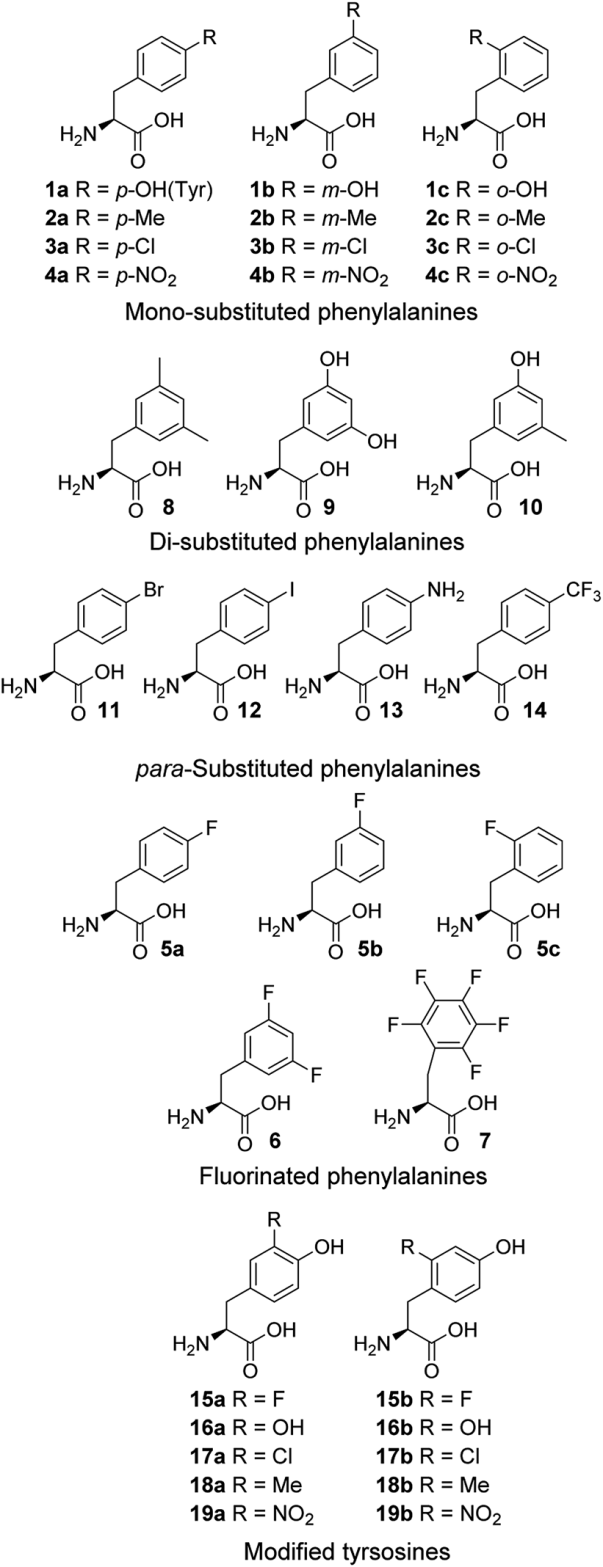
Amino acids used in this study.

Protein synthesis is a strictly regulated biosynthetic pathway. The remarkable substrate specificity exhibited by aminoacyl-tRNA synthetases (aaRSs),^1*a*^ a family of enzymes that catalyze the aminoacylation of amino acids with the cognate tRNA, is important for the high degree of accuracy.^1*a*^ However, examples of non-canonical amino acids mistakenly incorporated into proteins *via* a natural biosynthetic pathway have been reported,^2^ indicating that there is a limit to aaRS with regard to distinguishing substrates with structures slightly varied from the canonical amino acid. Here, studies probing the substrate range of wild-type *E. coli* phenylalanyl-tRNA synthetase (PheRS) and tyrosyl-tRNA synthetase (TyrRS) through the expression of protein with structural analogues of l-phenylalanine (Phe) and l-tyrosine (Tyr) (Fig. 1), respectively, are described. The two aaRSs are known for their ability to recognize cognate substrates that differ by only a hydroxyl group at the *para* position of the substrate phenyl ring.^1^ Some non-canonical amino acids display quite diverse physiological and pharmaceutical activity,^3,4^ whereas others are involved in many regards, such as the use for biological markers,^4^ altering protein properties^5^ and the production of new biological materials.^6^ Further, some hydroxylated derivatives are found to be the most abundant modified amino acids bound to proteins that are commonly detected in pathological tissues, and their misincorporation has been associated with a wide variety of pathological conditions such as aging,^7^ atherosclerosis,^4,8^ cataractogenesis,^4,9^ myocardial ischaemia and reperfusion^10,11^ and neurodegenerative disorders such as Parkinson's diseases.^12,26*a*^

The aminoacylation reaction catalyzed by aaRSs is the first step in protein synthesis and is accomplished *via* two stages.^1^ The amino acid first uses adenosine triphosphate (ATP) to form an aminoacyl-adenylate along with the release of pyrophosphate, followed by transfer of the amino acid to the terminal adenosine of the cognate tRNA.^1^ This has been identified as a rate determining step for both phenylalanyl-tRNA synthetase and tyrosyl-tRNA synthetase, with initial rates of transfer of the amino acid from aminoacyladenylate to cognate tRNA similar to the overall turnover number for the steady state aminoacylation of tRNA.^14*c*^ The aminoacylation is a key point in translational quality control as it provides the link between an amino acid, the tRNA anticodon and mRNA codon to enable accurate translation. Under physiological conditions, the estimated average error rate of amino acid misaminoacylation by aaRSs is 10^−4^.^13^ For some aaRSs, the accurate amino acid selection is accomplished mainly at their active sites, discriminating non-cognate amino acids on steric grounds. In other cases, there is an additional proofreading mechanism assisting the selection,^14^ which occurs at a site referred to as an “editing site”.^14*b*^ The latter is known as “double-sieve amino acid selection”.^14^ While protein synthesis using non-canonical amino acids is an indirect measure of the aaRS activity, work presented here was aimed to investigate the enzyme in a more direct manner, by evaluating the interactions between the non-canonical amino acids and the aaRS through an aminoacylation reaction.

Cell-free expression systems use a cell extract, which is partially purified and supplemented with the target DNA, amino acids and other ingredients, to enable *in vitro* protein synthesis in a controlled manner. The cell-free systems allow substitution of a standard amino acid with an unusual one, therefore can bias the competition in favor of the unusual amino acids. By this technique, incorporation of chloro,^15*a*,*b*^ dehydro^15*c*^ and hydroxyl^17*a*^ amino acids has been reported. Here, an *E. coli*-derived cell-free system was used to study the modified phenylalanines 1–14 and tyrosines 15–19 (Fig. 1). In these amino acid analogue systems the volume of the substituent on the phenyl ring of non-canonical amino acids is varying, and so is the hydrophobicity. We investigated how the size and hydrophobicity of substituent affect the incorporation of non-canonical amino acids. In earlier reports, translational incorporation of 5a–c into proteins as substitutes for Phe,^16^ and 15a, 16a, 17a and 19a for Tyr^17^ have been described. However, the incorporation levels of many of the amino acids and the extent to which they compete with the natural substrate have not been determined.

## Results and discussion

### *E. coli* cell-free protein synthesis with Phe analogues

It is known that PheRS is highly selective for Phe over Tyr.^1^ However, Tyr isomers *m*-Tyr (1b) and *o*-Tyr (1c), and well as 3,4-dihydroxyphenylalanine (3,4-DOPA, 16a) with two OH groups on the ring of the substrate, were reportedly activated^18*a*–*c*^ and incorporated^18*d*–*f*^ into proteins by PheRSs from various organisms. These suggest that, although PheRS has evolved to distinguish between Phe and Tyr primarily through recognition of the OH group at *para* position, it imparts far lower selectivity against the OH at other positions on the substrate ring.

**Fig. 2:**
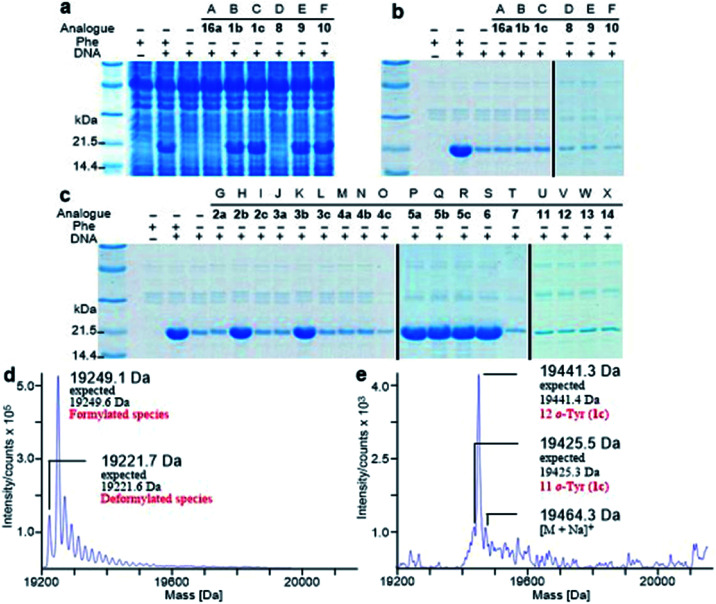
Analysis of synthesized His_6_-PpiB. (a) SDS-PAGE analysis before purification; (b and c) SDS-PAGE analysis after purification. Lanes A–X: no Phe but with 16a, 1b, 1c, 8, 9, 10, 2a, 2b, 2c, 3a, 3b, 3c, 4a, 4b, 4c, 5a, 5b, 5c, 6, 7, 11, 12, 13 or 14. (d and e) Mass spectral analysis of wild-type His_6_-PpiB and His_6_-PpiB synthesized in the presence of 1c.

*E. coli* peptidyl-Pro *cis*–*trans* isomerase B, which has been extensively studied,^19^ with a hexahistidine tag attached at the N-terminus (His_6_-PpiB) was expressed as a test protein. It contains 12 and 3 sites for Phe and Tyr, respectively.^19^ SDS-PAGE analysis of the synthesized His_6_-PpiB shows that protein was produced when all components required for protein synthesis were supplied (Fig. 2, with DNA and Phe). Mass spectral analysis (Fig. 2d) confirmed that it is wild-type protein, and corresponds to the pair of the predominant peaks. The presence of two signals in mass spectra is due to incomplete deformylation of PpiB by deformylase in the *E. coli* S30 extract.^17*a*^ By comparison, only a trace of protein was produced when Phe was excluded from the reaction mixture (Fig. 2, with DNA but no Phe). Mass spectral analysis (data not shown) showed that it is wild-type His_6_-PpiB, suggesting that the production of background protein was due to the presence of traces of Phe during cell-free reaction. This experiment also confirms that Tyr is not incorporated into His_6_-PpiB in place of Phe, as the reaction mixture contained a typical amount of Tyr (1.0 mM) for cell-free synthesis. No protein band corresponding to His_6_-PpiB was observed when the DNA plasmid was not added.

Protein expression was efficient when Phe was substituted with 1b,c (Fig. 2a, lanes B and C), reaching almost wild-type levels. Interestingly, the expressed proteins precipitated during purification (Fig. 2b, lanes B and C). This hinted at the possible effects the non-standard amino acid can have on protein structural stability, in particular given the abundance in the protein (12 Phe sites). To ensure that the tested amino acids were indeed incorporated into the protein, the small amounts of isolated protein were analyzed by mass spectrometry (Fig. 2e). Given the presence of peaks mainly corresponding to completely substituted protein species, the average level of substitution of 1c for Phe was concluded to be greater than 95%, whereas a 90% was given for 1b (ESI Fig. S1†). This incorporation level was assessed semiquantitatively, by following a method described in an earlier report,^17*a*^ through a comparison of the height of the peaks corresponding to 12, 11 and 10 substitutions in the mass spectrum (Fig. 2e). The amount of His_6_-PpiB produced with 16a (Fig. 2a and b, lane A) is comparable to that produced in the absence of Phe, suggesting that 16a does not incorporate instead of Phe. The mass spectra of this protein (data not supplied) showed wild-type His_6_-PpiB.

Subsequently, Phe derivatives containing Me, Cl and NO_2_ substituents including mono-substituted 2a–c, 3a–c, 4a–c and di-substituted 8–10, as well as *para*-substituted derivatives 11–14, were studied in an analogous manner. Of these, protein was only produced with the *meta* isoforms, including mono-derivatized 2b and 3b (Fig. 2c, lanes H and K), and di-derivatized 9 and 10 (Fig. 2a and b, lanes E and F), suggesting greater flexibility around that position at the synthetase active site. Interestingly, the proteins synthesized with 2b and 3b remained soluble and were successfully isolated without apparent loss of yield, whereas in the cases of 9 and 10, white precipitates were observed after the completion of reaction. Consequently, samples of 9 and 10 for mass spectral analysis were prepared with two methods, by chromatographic purification of the supernatant, which resulted in neither protein bands in SDS-PAGE nor signals in the expecting region in mass spectra, and by solubilizing the insoluble part with buffer containing a high concentration of urea, which showed a high ratio of signal-to-noise in the mass spectra (ESI Fig. S8 and S9†). This suggests that the insoluble part was mainly His_6_-PpiB containing 9 and 10. The expression was then repeated with derivatives bearing one or more fluorines on the aromatic ring, of which all but the pentafluorophenylalanine (7) were incorporated into the expressed His_6_-PpiB (Fig. 2c, lanes P–S). In all successful cases soluble proteins were obtained and the incorporation of intact amino acid was verified by mass spectrometry (ESI Fig. S4–S7†). Incorporation of the fluorides 5a–c as substitutes for Phe using whole cell-based methods has been reported previously.^16^ The results obtained here are consistent with previous studies and confirm that the incorporation will occur in a cell-free system.

**Fig. 3:**
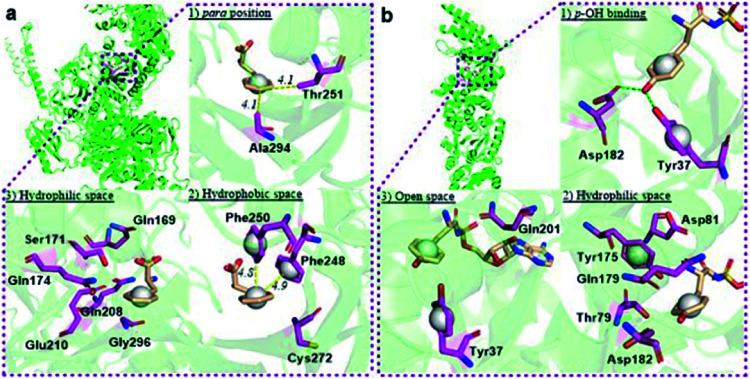
Structural analyses of *E. coli* PheRS and TyrRS. (a) *E. coli* PheRS synthetic active site with bound Phe;^27^ (b) *E. coli* TyrRS synthetic active site with bound tyrosyl-adenylate.^23^ The substrates are colored tan and amino acid residues purple. Yellow dashes measure distance between atoms. Green dashes represent hydrogen bond interactions. Round balls are placed in substrate rings to enhance views. Diagrams reproduced with the permission of Elsevier.

The reported crystal structure of the *E. coli* PheRS–Phe complex (Fig. 3a) indicates that the active site of *E. coli* PheRS is a mainly hydrophobic pocket with stringent steric requirements for the phenyl ring.^27^ The Me group of alanine at position 294 (Ala294) provides a steric constraint at the *para* position, which has been proposed as the primary factor assuring the high degree of selection of Phe over Tyr (Fig. 3a1). Previous mutagenesis studies have shown that substituting alanine with glycine (Ala294Gly) expands the volume of the active site and thus relaxes the substrate specificity of *E. coli* PheRS. As a consequence, Tyr and derivatives of Phe with *para*-substituents larger than an OH group, including Cl, Br, I, and ethynyl-, cyano- and azido-groups, are accepted and activated in aminoacylation reaction.^28^ In comparison, substitution with serine (Ala294Ser) decreases the volume and leads to a narrower substrate range for the engineered enzyme, rejecting even 5a.^28*a*,*b*^ The results of the present work correlate with these previous studies and support the role of synthetic pocket size as a determinant of specificity. The incorporation of 5a, and the lack of incorporation of the *para*-substituted derivatives 1a, 2a, 3a, 4a, 11–14, indicate slight flexibility at the active site of the wild-type *E. coli* PheRS near the *para* position, being able to accommodate only an F group. The requirements for the *ortho* and *meta* positions, on the other hand, are not so rigid and the synthetase has been shown to accept larger ring substituents. On one side of the ring the positions are observed projecting toward a small hydrophobic space (Fig. 3a2), surrounded by the side chains of Phe248 and Phe250 arranged in a “edge-to-face” manner for each aromatic pair, whereas the corresponding positions on the opposite side are enclosed with the side chains of Ser171, Gln174, Gln208, Glu210 and Gly296, pointing to a hydrophilic area (Fig. 3a).^27^ Incorporation of 5b,c in place of Phe suggests acceptance of an F at each position, which might have been expected given the flexibility of the *para* position. In addition to an F, an OH was also accepted at the *ortho* position, as suggested by the results with 1c, and an OH, Me and Cl at the *meta* position by the results with 1b, 2b and 3b. Further evidence by di-substituted amino acids 6, 9 and 10 reveals difference of the *meta* positions on the two ring sides, with one being able to accept a group as large as an Me and the other an OH.

To assist the selection for Phe, there is also an editing site located in the B3/4 domain of the β-subunit, about 35 Å from the synthetic active site, providing a post-transfer proofreading mechanism through hydrolysis of the tRNA^Phe^ mistakenly charged with non-cognate amino acid.^27^ However, this proofreading activity appears to require a phenol OH for hydrogen bond formation with the side chain of Glu334 and the main chain amide of Gly315.^27^ Previous reports have shown that *E. coli* PheRS hydrolyzes the tRNA^Phe^ misacylated with Tyr^1*b*^ and 1b,^22^ but does not cleave the tRNA^Phe^ charged with *para*-substituted (fluoro-, chloro-, bromo- and amino-) Phe derivatives,^28^ further evidence comes from incorporation of 5a*via* wild-type machinery,^16,28*a*,*b*^ as discussed above, and of *p*-Cl-Phe (3a) using a Ala294GlyPheRS mutant-containing *E. coli* strain.^28*b*^ Consistent with these, results of the current work support the requirement for hydrogen bond interactions and showed translational incorporation of a series of amino acid derivatives. Of particular interest was a mutant with significantly reduced editing activity, by which formation of the *m*-Tyr-tRNA^Phe^ was successful in aminoacylation.^22^ This indicates that 1b is a substrate of both the active and editing sites of *E. coli* PheRS, but the rate of hydrolysis is far lower than the rate of activation. This may account for the incorporation of 1b, and that of the 1c, 9 and 10 with at least one OH group on the substrate ring.

### *E. coli* cell-free protein synthesis with Tyr analogues

**Fig. 4:**
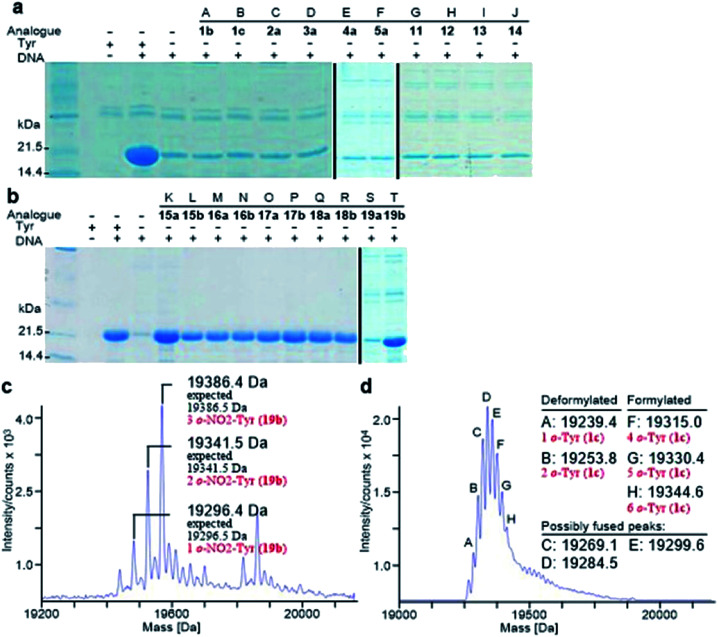
Analysis of synthesized His_6_-PpiB. (a and b) SDS-PAGE analysis of synthesized His_6_-PpiB. Lanes A–T: no Tyr but with 1b, 1c, 2a, 3a, 4a, 5a, 11, 12, 13, 14, 15a, 15b, 16a, 16b, 17a, 17b, 18a, 18b, 19a or 19b. (c) Mass spectral analysis of His_6_-PpiB synthesized in the presence of 19b; (d) mass spectral analysis of His_6_-PpiB synthesized in the presence of 0.2 mM Phe and 2.0 mM 1c. In (d), the peaks at 19 239.4 Da (A) and 19 253.8 Da (B) correspond to the respective 1 and 2 replacements of Phe residues for deformylated His_6_-PpiB, and peaks at 19 315.0 Da (F), 19 330.4 Da (G) and 19 344.6 Da (H) to the respective 4, 5 and 6 replacements of Phe residues for formylated His_6_-PpiB. The peaks at 19 269.1 Da (C), 19 284.5 Da (D) and 19 299.6 Da (E) are probably a result of fused peaks of the substituted deformylated and formylated His_6_-PpiB.

Analogously, the substrate scope of *E. coli* TyrRS was studied through protein expression with Tyr analogues. To assess the requirement for the ring location of the OH, Tyr isomers 1b,c were studied. To assess the importance of the *p*-OH group, derivatives bearing a range of *para*-substituents, including 2a, 3a, 4a, 5a, 11–14 were studied. None of these amino acids were able to support protein expression (Fig. 4a).

Having established that *p*-OH is not replaceable for substrate recognition, Tyr derivatives with substituents at other ring positions, including both *ortho* and *meta* positions, were investigated. Derivatives studied involve the fluorides 15a,b, and hydroxyl-, chloro-, methyl- and nitro-substituted 16a,b, 17a,b, 18a,b and 19a,b. Apart from 19a, soluble proteins were obtained and successfully isolated with all derivatives (Fig. 4b), even with 16a,b that bear two OH groups. Compared to protein loss observed for 1b,c, successful isolation with 16a,b is probably attributable to a low level of abundance in the protein (3 Tyr sites). With a similar cell-free system, incorporation of 19a in place of Tyr was reported.^17*d*^ It was accomplished by considerably enhancing the concentrations of *E. coli* TyrRS, which was prepared separately and supplemented to the system exogenously, and the substrate 19a.^17*d*^ The necessity for this suggests a poor activation by *E. coli* TyrRS, which is most probably the cause of the lack of incorporation in the present study.

The reported crystal structure of the *E. coli* TyrRS–tyrosyl-adenylate complex (Fig. 3b) shows that the enzyme forms hydrogen bonds with the *p*-OH group of Tyr by the side chains of Tyr37 and Asp182 (Fig. 3b).^23^ The *p*-OH group is a key element for the substrate specificity of *E. coli* TyrRS as it offers complementary hydrogen bonds for the recognition of the amino acid,^29^ that of the twenty normal amino acids only Tyr can form. Studies of the Tyr isomers and *para*-substituted derivatives show that this OH group must locate at the *para* position and it is not replaceable. In analogy to the *E. coli* PheRS active site, spaces near ring positions are observed for *E. coli* TyrRS.^23^ The *ortho* and *meta* positions on one side of the substrate ring, enclosed by the side chains of Tyr175, Thr76 and Gln179, project to a small hydrophilic space (Fig. 3b), whereas the corresponding positions on the other side point to an open area (Fig. 3b), in particular the *ortho* position. Consistent with earlier findings,^17*a*–*c*^ results obtained with 15a, 16a and 17a indicate the space near the *meta* position at the *E. coli* TyrRS active site can accommodate an F, OH or Cl. In addition, this space has been shown to accommodate a Me (18a). The *ortho* position displayed the most flexibility, being able to accept not only an F, OH, Cl and Me but also a NO_2_.

### Competition between the cognate and derivatized amino acids

A major advantage of the cell-free expression system over whole cell-based techniques is to precisely control the concentration of substrate. This allows to determine the extent to which the non-canonical amino acids compete with the natural counterpart. Competitive experiments between Phe and Tyr and their related derivatives were therefore performed. Although many amino acid analogues were found to be efficient substrates of *E. coli* PheRS or TyrRS, the natural amino acids were highly favoured against all analogues investigated (Table 1). Inclusion of a small amount of Phe in the reaction mixture precluded the incorporation of most Phe analogues. The inefficient competition of only 1c (2.1%) and the fluorides 5a–c (3.6%, 2.1% and 1.4%, respectively, Table 1) shows that the selectivity for Phe is at least two orders of magnitude higher than for all surrogates. The *K*_M_ value of *E. coli* PheRS for Phe (0.05 mM) is reported to be approximately 100-, 30- and 10-fold lower than that for 5c (5.0 mM), 5b (1.4 mM) and 5a (0.56 mM),^20^ respectively (Table 1). These ratios correlate with the competition efficiencies obtained. In an analogous manner, preclusion of most Tyr analogues was also observed with a small amount of Tyr in the reaction mixture. The inefficient competition of 15a,b (2.9% and 2.3%, respectively, Table 1) suggests that the selectivity for Tyr is at least two orders of magnitude higher. The ratio of the *K*_M_ value of *E. coli* TyrRS for Tyr (0.0061 mM)^21^ relative to that of 15a (0.13 mM)^21^ (4.7%) also correlates with the competition efficiencies obtained for 15a.

Summary of incorporation levels and competition efficiencies of Phe and Tyr derivatives, and kinetics of *E. coli* TyrRS

**Table:** 

Derivative (2.0 mM)	Incorporation level^a^ (%)		Competition efficiency (%)	*k*_cat_ a (s^−1^)	*K*_M_ (mM)	*k*_cat_/*K*_M_ (s^−1^ M^−1^)
	No Phe	0.2 mM Phe				
Phe	—	—	—	—	0.05(ref. 20)	—
1b	90	N.D.	N.D.^b^	2.1 (ref. 22)	0.25 (ref. 22)	0.8 × 10^4^
1c	>95	21 ± 0	2.1	—	—	—
2b	>95	N.D.	N.D.	—	—	—
3b	>95	N.D.	N.D.	—	—	—
5a	>95	36 ± 0.9	3.6	—	0.56 (ref. 20)	—
5b	>95	21 ± 1.7	2.1	—	1.4 (ref. 20)	—
5c	>95	14 ± 2	1.4	—	5.0 (ref. 20)	—
6	>95	N.D.	N.D.	—	—	—
9	>95	N.D.	N.D.	—	—	—
10	>95	N.D.	N.D.	—	—	—

**Table:** 

Derivative (2.0 mM)	Incorporation level^a^ (%)		Competition efficiency (%)	*k*_cat_ a (s^−1^)	*K*_M_ (mM)	*k*_cat_/*K*_M_ (s^−1^ M^−1^)
	No Tyr	0.2 mM Ty				
1a (Tyr)	—	—	—	11.3 ± 0.15	0.006 (ref. 21)	1.85 × 10^6^
15a	>95	29 ± 0.6	2.9	6.5 ± 0.14	0.13 (ref. 21)	5.02 × 10^4^
15b	>95	23 ± 1.2	2.3	4.8 ± 0.12	—	—
16a	>95	N.D.	N.D.	3.3 ± 0.01	1.4 (ref. 21)	2.33 × 10^3^
16b	>95	N.D.	N.D.	0.89 ± 0.01	—	—
17a	>95	N.D.	N.D.	0.34 ± 0.07	—	—
17b	>95	N.D.	N.D.	0.17 ± 0.08	—	—
18a	∼90	N.D.	N.D.	0.09 ± 0.07	—	—
18b	>95	N.D.	N.D.	0.13 ± 0.06	—	—
19a	<10	N.D.	N.D.	N.D.	—	—
19b	∼80	N.D.	N.D.	0.06 ± 0.01	—	—

a Incorporation levels and rates of reaction determined in duplicate experiments varied by less than 20%. Data are the mean of duplicate experiments.

b N.D. = not determined.

### Monitoring activity of *E. coli* TyrRS

Apart from the protein expression experiments, the interactions of the tested analogues with the *E. coli* TyrRS were assessed directly through adenosine monophosphate (AMP) production assays. Of the two synthetases indirectly investigated by protein expression, *E. coli* TyrRS was selected as it only has one active site.^23^ From Table 1 it is evident that *E. coli* TyrRS catalyzes the reactions of Tyr analogues at different rates, suggesting varying degrees of binding of the synthetase toward each analogue. Among all substrates, a highest *k*_cat_ value was obtained for Tyr, whereas the reaction with Phe was not detected. This is expected as Tyr is the natural substrate while Phe is not. No reaction with 19a was observed either, despite that an earlier report has described the detection of aminoacylation of tRNA^Tyr^ with 19a by *E. coli* TyrRS using a mass spectrometry-based technique.^25^ As shown in control experiment (Fig. 4c), 0.5 μM AMP is in the limit of quantification by HPLC. This suggests that the AMP produced with 19a was below detection limit. As mentioned above, it is probably a result of poor activation by the enzyme. Attempts to enhance the concentrations of components in the present study to measure the reaction rate were unsuccessful, due to the poor solubility of 19a.

In view of the *k*_cat_ values of the *meta*-substituted tyrosines 15a, 16a, 17a and 18a, it appears that the relative reaction rate decreases in relation to an increase of van der Waals radius of the substituent on the aromatic ring of each analogue. The same pattern was also observed for the *ortho*-substituted species 15b, 16b, 17b, 18b and 19b. This shows that *E. coli* TyrRS distinguishes small structural changes and catalyzes reaction of each analogue at a varying rate. A combination of the *k*_cat_ for Tyr and its *K*_M_ value affords a catalytic efficiency (*k*_cat_/*K*_M_) being the greatest among the three ligands with reported *K*_M_ values (Tyr itself, 15a and 16a) (Table 1). A comparison of the catalytic efficiency of Tyr with 15a shows that 15a is activated only 2.7% as efficiently as Tyr by *E. coli* TyrRS. This ratio correlates with the 2.9% competition efficiency observed in competitive experiment (Table 1), indicating that competing for incorporation is essentially a competition for the activation by *E. coli* TyrRS. These show a direct relationship between the ability of the synthetase to bind different substrates and catalyze the aminoacylation reactions at varying rates with the incorporation of non-canonical amino acids.

**Fig. 5:**
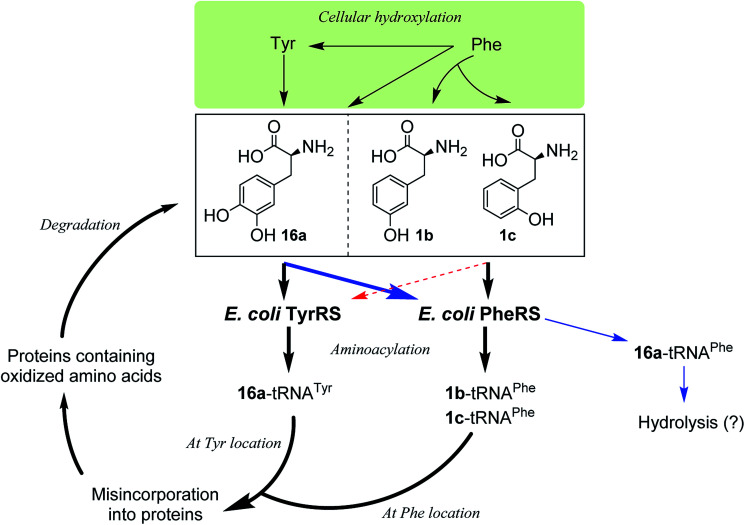
Proposed oxidized protein synthetic pathways by incorporation of oxidized amino acid *via E. coli* PheRS and TyrRS. Intracellular oxidation of Phe and Tyr produces noncanonical hydroxylated derivatives that are substrates of *E. coli* PheRS and TyrRS. 3,4-DOPA (16a) is a substrate for both PheRS and TyrRS, while *p*- and *m*-tyrosine (1b,c) are both substrates of PheRS. The proofreading domain of PheRS is likely to prevent incorporation of 16a into protein in place of Phe, however misincorporation of 16a in place of Tyr may occur along with misincorporation of 1b or 1c in place of Phe.

Incorporation of hydroxylated amino acids during protein synthesis has been associated with deposition and accumulation of oxidized proteins,^18*e*^ as in biological systems the aromatic amino acids Phe and Tyr are prone to modification by cellular oxidants such as reactive oxygen species to give the corresponding products 1b,c and 16a.^4,18*e*,26^ Elevated levels of the three amino acids have been found bound to oxidatively damaged proteins that are commonly detected in pathological tissues.^12,18*e*^ Although the involvement of aaRSs in this process has been hypothesized, the specific pathway by which oxidized amino acids are incorporated remains poorly understood. While both whole-cell and cell-free,^18*d*–*f*^ as well as aminoacylation^18*a*–*c*^ studies have shown that 1b,c could be incorporated *via* PheRS and 16a*via* TyrRS,^6,17*a*^ the incorporation may occur by the other way around, with 16a*via* PheRS and 1b,c*via* TyrRS, since activation of 16a by both bacterial and eukaryotic PheRSs has been observed,^18*b*,*c*^ as mentioned above and, as isomers of Tyr, 1b,c could in principle replace Tyr. In this work, we have clarified that, in an *E. coli* system, 1b,c can only be incorporated at the Phe location, not at the Tyr location, and that 16a would only be incorporated at the Tyr location (Fig. 5). This may aid the study of age-related pathologies associated with oxidized protein accumulation.

## Conclusions

The work described here shows inherent substrate promiscuity of the *E. coli* PheRS and TyrRS. They can activate a range of structural analogues and subsequently charge them onto the cognate tRNA for protein expression. To the best of our knowledge, it is the first time incorporation of the amino acid analogues 2b, 3b, 6, 9, 10, and 15b, 16b, 17b, 18 and 19b into protein *via* wild-type translational machinery has been observed.

The inherent substrate promiscuity of aaRS may prove useful as new tools for biotechnology, as it allows the production of proteins engineered with nonproteinogenic amino acids in a direct manner, without the need for aaRSs with relaxed substrate specificity. Unlike the case of expanded genetic codes, this system does not accommodate an additional noncanonical amino acid, instead replacing a Phe or Tyr partially or completely. There is no site specificity with regard to partial replacement and hence a plasmid cannot be generated to direct modifications in a predetermined position of the protein it encodes. In this work, the incorporation levels of most amino acids are greater than 95% and thus enabled a high degree of amino acid modification, including fluorination, oxidation, methylation, chlorination and nitration, which can not only be used for applications such as isotopic labeling for use in spectroscopic studies, but also for investigation of general rather than specific effects of amino acid derivatives on protein structure and function. In addition, this study also identifies the oxidized protein synthetic pathways by incorporation of oxidized amino acids involving aaRSs. Further, the substrate promiscuity of bacterial PheRS and TyrRS may be different from that of eukaryotic enzymes, making these two synthetases potential targets for the development of antibacterial agents.

## Experimental

### Syntheses

Most of the amino acid derivatives used in this study were commercially available, except for 8–10, 15b, 16b, 17b, 18 and 19b, which were synthesized in the laboratory. Apart from 3-hydroxy-5-methylphenylalanine (10), the preparation of the rest of the derivatives was described previously.

#### Diethyl 2-acetamido-2-(3-methoxy-5-methylbenzyl)malonate

To a solution of metallic sodium (0.235 g, 0.01 mol) in dry ethanol (20.0 mL), diethyl acetaminomalonate (2.17 g, 0.01 mol) was added. The resultant mixture was stirred at room temperature for 30 minutes, mixed with 3-bromomethyl-5-methylanisole (2.0 g, 0.0093 mol), heated under reflux for two hours, concentrated and extracted with ethyl acetate (3 × 5 mL). The combined extracts were washed with saturated brine, dried (sodium sulfate), filtered and concentrated, yielding a residue which was recrystallized from ethyl acetate and hexane to give diethyl 2-acetamido-2-(3-methoxy-5-methylbenzyl)malonate as a white solid (2.42 g, 74% yield). Mp 109.0–110.1 °C; ^1^H NMR (400 MHz, CDCl_3_): *δ* 6.75–6.79 (m, 1H; Ar H), 6.67–6.69 (m, 1H; Ar H), 6.53 (s, 1H; Ar H), 4.25 (q, *J* = 7.2 Hz, 4H; (C*H*_2_CH_3_)_2_), 3.77 (s, 3H; OC*H*_3_), 3.53 (s, 2H; Ar-C*H*_2_), 2.18 (s, 3H; OC*H*_3_), 2.01 (s, 3H; (C=O)C*H*_3_), 1.28 (t, *J* = 7.2 Hz, 6H; (CH_2_C*H*_3_)_2_). ^13^C NMR (100 MHz, CDCl_3_): *δ* 169.03 (NH*C*=O), 167.69 (2C, (*C*=O)OEt), 156.97 (Ar *C*), 132.26 (Ar *C*), 128.07 (Ar *C*), 126.67 (Ar *C*), 126.29 (Ar *C*), 109.74 (Ar *C*), 67.43 (quaternary C), 62.60 (2C, *C*H_2_CH_3_), 55.27 (O*C*H_3_), 37.03 (Ar-*C*H_2_), 23.09 ((C=O)*C*H_3_), 16.25 (Ar-*C*H_3_), 14.10 (2C, CH_2_*C*H_3_). HRMS (ESI) calcd for C_18_H_25_NO_6_Na [M + Na]^+^*m*/*z* 374.1580; found 374.1580. Calcd for C_18_H_26_NO_6_ [M + H]^+^*m*/*z* 352.1760; found 352.1763. Anal. calcd for C_18_H_25_NO_6_: C 61.53, H 7.17, N 3.99; found: C 61.31, H 7.17, N 4.12.

#### (*R*,*S*)-3-Hydroxy-5-methylphenylalanine (10)

A solution of diethyl 2-acetamido-2-(3,5-dimethoxybenzyl)malonate (2.0 g, 0.0057 mol) in 62% aqueous hydrobromic acid (11.5 mL) and acetic acid (7.0 mL) was heated in a sealed tube at 150 °C for two hours. The mixture was diluted with water (10.0 mL), decolorized with charcoal and concentrated, affording a residue which was recrystallized from water and ethanol to give (*R*,*S*)-3-hydroxy-5-methylphenylalanine as a white solid (0.89 g, 80% yield). Mp 250.0–253.8 °C. ^1^H NMR (400 MHz, D_2_O): *δ* 6.36 (s, 1H; Ar H), 6.31–6.32 (m, 2H; Ar H), 3.44–3.46 (m, 1H; C*H*), 2.86–2.90 (m, 1H; C*H*HCH), 2.56–2.62 (m, 1H; CH*H*CH), 2.19 (s, 3H; C*H*_3_). ^13^C NMR (100 MHz, D_2_O): *δ* = 171.21 (*C*=O), 153.17 (Ar-*C*), 131.93 (Ar-*C*), 127.87 (Ar-*C*), 125.60 (Ar-*C*), 125.51 (Ar-*C*), 115.47 (Ar-*C*), 53.99 (*C*H), 34.60 (*C*H_2_), 15.01 (*C*H_3_). HRMS (ESI) calcd for C_10_H_13_NO_3_Na [M + Na]^+^*m*/*z* 218.0793; found 218.0793. Anal. calcd for C_10_H_13_NO_3_: C 61.53, H 6.71, N 7.17; found: C 61.87, H 6.96, N 7.26.

### Construction of plasmids

The gene in pND706 vector encoding the *E. coli* His_6_-TyrRS was kindly provided by the Dixon group at the University of Wollongong (Australia). The genes in pND1098 vector encoding *E. coli* His_6_-PpiB and in pND706 vector encoding the *E. coli* His_6_-TyrRS were transformed into the respective *E. coli* DH5α and *E. coli* AN1459 for amplification and were isolated using Qiagen® Mini kit following procedures recommended by the manufacturer. The gene encoding the *E. coli* TyrRS was then cloned in pETMCSIII vector within the Ndel-HindIII sites to generate the plasmid pET-TyrRS-His. The sequence of pET-TyrRS-His was confirmed using ABI 3730 Genetic Analyzer. The concentration of the DNA was measured using a NanoDrop spectrophotometer.

### Preparation of *E. coli* S30 extract

The *E. coli* S30 extract was prepared from *E. coli* star BL21(DE3) by following a protocol established earlier.^15,17*a*^

### Cell-free protein synthesis

The *E. coli* cell-free protein synthesis was conducted by following a procedure, as described in earlier reports.^15,17*a*^ The natural amino acids were used at 1.0 mM. The unnatural amino acids were used at 2.0 mM and the corresponding natural amino acid was not added. The reactions were proceeded for 6 h at 30 °C, with shaking at 200 rpm, unless otherwise stated. The competitive experiments between unnatural amino acids and their corresponding natural counterparts were conducted in a system identical to that used for usual cell-free protein synthesis, with the cognate amino acid supplied to the expression at 0.2 mM concentration to compete with the foreign substrate at 2 mM. In cases where racemic mixtures were used, this concentration refers to the l-enantiomer content. The levels of unnatural amino acid incorporation in competitive experiments were concluded from the mass spectra. The competition efficiencies are given as percentages and were calculated based on the ratio of the concentrations between the natural amino acid and its corresponding competitor, and the level of unnatural amino acid incorporation (incorporation level (%) × [natural amino acid]/[derivative]). The amino acid derivatives that did not incorporate were excluded from this assay, except for 19a, which was studied for the purpose of comparison.

### Protein analysis

The crude contents of the inner reaction was analyzed by SDS-PAGE, to confirm the production of soluble protein. The inner reaction contents were then purified using the HisGraviTrap® Kit following the native conditions recommended by the manufacturer, and the resulting elution fraction was concentrated using Amicon Ultra-4 (YM-3, 000) centrifugal filter devices. Protein concentrations were measured using a NanoDrop. The mass of the protein produced was confirmed by the Agilent 1100 series LC/MSD TOF instrument.

### *E. coli* His_6_-TyrRS preparation and activity assay

**Fig. 6:**
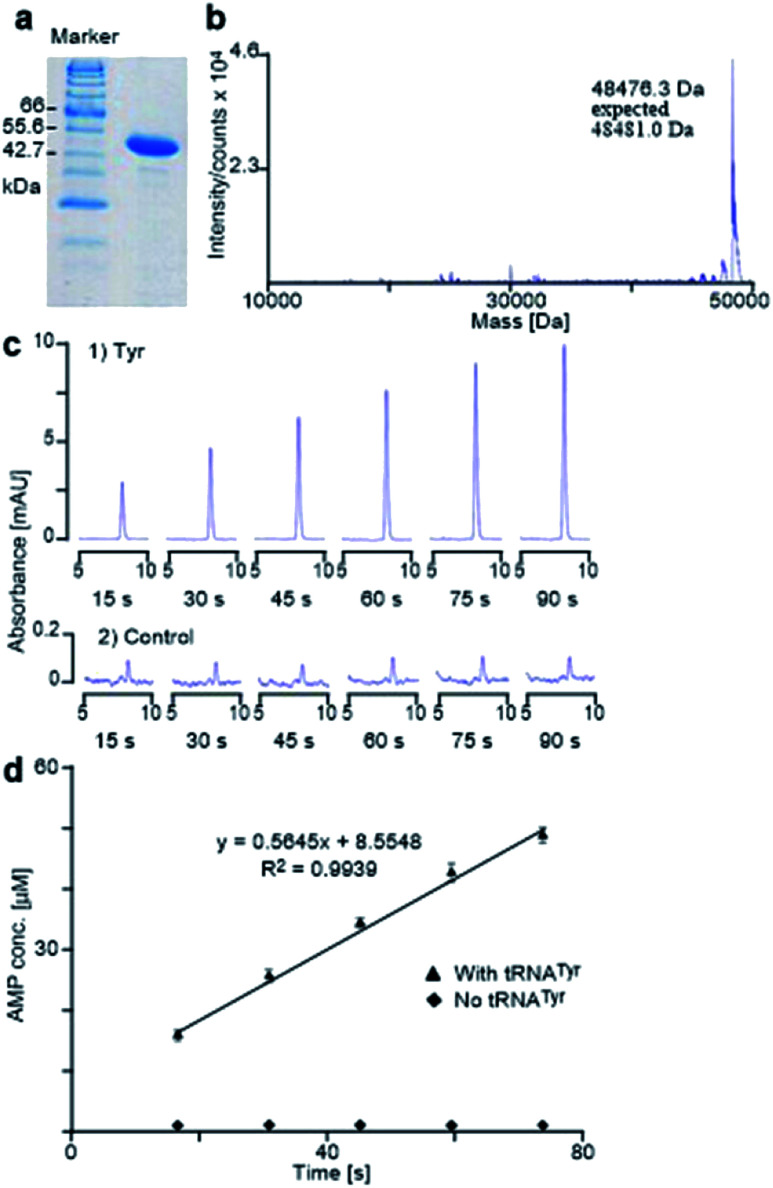
*E. coli* His_6_-TyrRS activity assay. (a and b) SDS-PAGE and mass spectral analyses of prepared *E. coli* TyrRS. (c) Partial HPLC traces of aliquots collected every 15 seconds from the reactions of Tyr (1) and the control experiment (2) that contained no tRNA^Tyr^. (d) The plot of the concentration of AMP against time for the aminoacylation of Tyr. Data points are the mean of duplicate experiments, with errors bars representing ± SD.

The *E. coli* TyrRS with a polyhistidine tag at the C-terminus (*E. coli* His_6_-TyrRS) was overproduced from pET-TyrRS-His in *E. coli* BL21 (DE3). The resulting enzyme was purified using HisGraviTrap® Kit following the conditions recommended by the manufacturer, and was concentrated using Amicon Ultra-4 (YM-3, 000) centrifugal filter devices. The prepared enzyme was analyzed by SDS-PAGE (20% acrylamide) with mass confirmed by Agilent 1100 series LC/MSD TOF instrument (Fig. 6a and b). The enzyme concentration was measured using a NanoDrop spectrophotometer. The method used to evaluate the activity of the prepared enzyme determines the catalytic rate constant (*k*_cat_) through HPLC monitoring the formation of AMP by analyzing aliquots taken from the reaction mixture and quenched with 0.1% SDS every 15 seconds (Fig. 6c). It gave Tyr a *k*_cat_ of 11.3 s^−1^ (Fig. 6c and d), which is in excellent agreement with reported value of 12 s^−1^.^24^

### Aminoacylation assay

The aminoacylation of amino acid derivative was conducted in a manner identical to Tyr. Stock solutions were prepared in Tris–HCl buffer containing 100.0 mM Tris, 15.0 mM MgCl_2_, 40.0 mM KCl and 1.0 mM DTT, at pH 7.6. The reaction was performed at 37 °C in a 0.5 mL microcentrifuge, with a total volume of 60 μL containing 0.05 or 0.15 μM *E. coli* His_6_-TyrRS, 1.0 mM ATP, 16.7 or 60.0 μM tRNA^Tyr^, and 0.1 mg mL^−1^ bovine serum albumin (BSA). After pre-incubation for one minute, the reaction was initiated by adding in the *E. coli* His_6_-TyrRS. A 10 μL sample was taken out by hand from the proceeding reaction every 15 or 40 seconds and quenched into 5.0 μL 0.3% (w/v) aqueous sodium dodecylsulfate (SDS). The SDS-treated samples were either immediately analyzed by HPLC or stored at −20 °C for later analysis. In the latter case, the storage was never longer than 12 hours. The solvent system used for HPLC analysis was as follows:

**Table:** 

Time (min)	Buffer A (%)	Buffer B (%)
0	87	13
18	87	13
19	70	30
23	70	30
24	87	13

In this solvent system, Buffer A contains 60.0 mM ammonium phosphate and 5.0 mM tetrabutylammonium phosphate, at pH 5.0, while Buffer B contains 5.0 mM tetrabutylammonium phosphate in methanol. Both buffers were filtered and degased before use.

## Conflicts of interest

There are no conflicts to declare.

## Supplementary Material

RA-010-D0RA00655F-s001
