# Supplementary material for: Translational incorporation of modified phenylalanines and tyrosines during cell-free protein synthesis
Source: RSC Adv. 2020 Mar 18;10(19):11013–23. doi: 10.1039/d0ra00655f (PMC9050441; doi:10.1039/d0ra00655f)
Supplement: RA-010-D0RA00655F-s001 [file RA-010-D0RA00655F-s001.pdf]

Electronic Supplementary Material (ESI) for RSC Advances.
This journal is © The Royal Society of Chemistry 2020

SUPPORTING INFORMATION FOR:

Translational Incorporation of Modified Phenylalanines and Tyrosines During Cell-free Protein Synthesis

Zhongqiang Wang[1,2,3]* and Hayden Matthews[4]*

[1]*Key Laboratory of Biocatalysis & Chiral Drug Synthesis of Guizhou Province, Generic Drug Research Center of Guizhou Province, School of Pharmacy, Zunyi Medical University, Zunyi, 563000, China.*

[2]*Key Laboratory of Basic Pharmacology of Ministry of Education and Joint International Research Laboratory of Ethnomedicine of Ministry of Education, Zunyi Medical University, Zunyi, 563000, China.*

[3]*Research School of Chemistry, Australian National University, 137 Sullivans Creek Road, Acton ACT 2601 Australia*

[4]*Australian National University Medical School, 54 Mills Road, Acton ACT 2601 Australia*

zqwang@zmu.edu.cn, Hayden.Matthews@anu.edu.au

CONTENTS **PAGE**

1. General Experimental 2

2. Syntheses 2

3. Compound characterization 15
Diethyl 2-acetamido-2-(3-methoxy-5-methylbenzyl)malonate
(*R*,*S*)-3-hydroxy-5-methylphenylalanine (**10**)

4. ESI Mass spectrometry of purified proteins 22

5. Representative HPLC of the aminoacylation of tyrosine 32

6. References 33

1. General Experimental

Structural analogues of Tyr and Phe, and chemicals for synthesis were purchased from Sigma-Aldrich Co., PepTech Co., Alfa Aesar, Manchester Organics, Matrix Scientific, Oakwood Products and Toronto Research Chemicals. They were of commercial quality and were used as received, apart from the (*R,S*)-3-fluorotyrosine and (*S*)-3-chlorotyrosine (Aldrich) that were purified by HPLC. The genes in pND1098 vector encoding *E. coli* His_6-PpiB and in pND706 vector encoding the *E. coli* His_6-TyrRS were generous gifts of the Dixon group at the University of Wollongong, Wollongong, New South Wales, Australia. T7 RNA polymerase (50 000 $U.mL^{-1}$) was purchased from New England BioLabs. The ammonium phosphate, tetrabutylammonium phosphate in methanol and *E. coli* $tRNA^{Tyr}$ were purchased from Sigma-Aldrich Co. Spectra/Por dialysis membrane (#2, MWCO: 12-14 000) was purchased from Spectrum Laboratories. Mini-PROTEAN® Tetra Electrophoresis System, acrylamide, bis-acrylamide, SDS-PAGE molecular weight standards (low and broad range) and Bio-Safe® Coomassie Blue were purchased from Bio-Rad. HisGraviTrap® Kit was purchased from GE Healthcare. Amicon Ultra-4 (YM-3,000) membrane microcon centrifugal filter devices were purchased from Millipore.

^{1}H and ^{13}C NMR spectra were recorded on a Varian MR400 or a Varian Mercury 300 instrument. ESI MS were obtained using a Micromass VG AutoSpec M spectrometer. Elemental analyses were performed using a Eurovector EA3000 Elemental Analyser. Melting points were determined using a Kofler hot-stage melting point apparatus under a Reichert microscope. Mass spectra of biological macromolecules were recorded on an Agilent 1100 series LC/MSD TOF instrument (direct injection) operating a positive ionization mode, with the liquid chromatography running a mobile phase of a 50:50 (v/v) solution of 0.1% formic acid in acetonitrile: 0.1% aqueous formic acid. The mass spectra were recorded and then deconvoluted using the Agilent TOF Protein Confirmation Software. All mass spectra presented here were those after deconvolution. Gene sequencing was conducted using the ABI 3730 Genetic Analyser in conjunction with BigDye 3.1 at The Australian National University, Canberra. The concentrations of protein and DNA were measured using a NanoDrop ND-1000 spectrophotometer (ThermoScientific). SDS-PAGE analyses of proteins were conducted with 20% acrylamide.

For purification, HPLC was carried out on a Waters 600 controller with Waters 717plus autosampler and a Waters 2996 photodiode array detector, running Empower 2 software. For aminoacylation assay, HPLC was carried out using Agilent Hewlett Packard series 1100 separation system operating on Chemstation, in conjugation with the Alltech Alltima HP C^{18} 5 mM (4.6 x 250 mm) column attached with an Alltech Allguard HP C^{18} guard cartridge.

2. Syntheses

1-(Bromomethyl)-3,5-dimethylbenzene

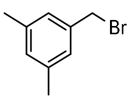

1-(Bromomethyl)-3,5-dimethylbenzene was prepared from mesitylene by the method of Dailey.[1] The choice of α,α,α-trifluorotoluene instead of carbon tetrachloride as the solvent for bromination is for environmental reasons and is based on the work of Golding.[2] To a solution of mesitylene (2.0 g, 0.017 mol) in anhydrous α,α,α-trifluorotoluene (30.0 mL), *N*-bromosuccinimide (2.94 g, 0.017) and azobisisobutyronitrile (0.09 g, 0.00055 mol) were added. The

resultant mixture was heated under reflux for one hour, then filtered and concentrated, yielding a residue which was purified with chromatography from chloroform and hexane to give 1-(bromomethyl)-3,5-dimethylbenzene as a pale yellow semi-solid at room temperature (lit.[3] mp 37.5−38 °C). Yield 1.72 g, 52%. ^{1}H NMR (400 MHz, $CDCl_3$): δ 7.07 (s, 2H; Ar H), 6.99 (s, 1H; Ar H), 4.49 (s, 2H; CH_2), 2.37 (s, 6H; $(CH_3)_2$); ^{13}C NMR (101 MHz, $CDCl_3$): δ 138.44 (2C, Ar *C*), 137.67 (Ar *C*), 130.26 (Ar *C*), 126.91 (2C, Ar *C*), 34.00 (CH_2), 21.27 (2C, CH_3). MS (ESI): *m/z* (%): 119.0 (C_9H_{11}) (100), 199.2 ($[M + H]^+$) (30). The spectral properties of this compound were identical with those previously reported for 1-(bromomethyl)-3,5-dimethylbenzene.[4]

Diethyl 2-acetamido-2-(3,5-dimethylbenzyl)malonate

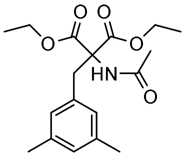

Diethyl 2-acetamido-2-(3,5-dimethylbenzyl)malonate was prepared from 1-(bromomethyl)-3,5-dimethylbenzene by the method used for alkylation of 3,5-dimethoxybenzyl bromide.[5] A solution of metallic sodium (0.17 g, 0.0075 mol) and diethyl acetaminomalonate (1.627 g, 0.0075 mol) in dry ethanol (20.0 mL) was stirred at room temperature for 15 minutes. To the resultant mixture, 1-(bromomethyl)-3,5-dimethylbenzene (1.5 g, 0.0075 mol) was added, followed by heating under reflux for three hours, and filtered. Then water (70.0 mL) was added to the filtrate, affording a separation of an oil which crystallized upon cooling, giving diethyl 2-acetamido-2-(3,5-dimethylbenzyl)malonate as white powder. Yield 2.51 g, 55%. mp 149.6−152.3 °C (lit.[1] mp 149−150 °C). ^{1}H NMR (400 MHz, $CDCl_3$): δ=6.85 (s, 1H; N*H*), 6.59 (s, 2H; Ar H), 6.54 (s, 1H; Ar H), 4.25 (q, *J* = 7.2 Hz, 4H; $(CH_2CH_3)_2$), 3.55 (s, 2H; Ar-CH_2), 2.23 (s, 6H; $(Ar\text{-}CH_3)_2$), 2.01 (s, 3H; (C=O)CH_3), 1.28 (t, *J* = 7.2 Hz, 6H, $(CH_2CH_3)_2$). ^{13}C NMR (101 MHz, $CDCl_3$): δ=169.04 (NH*C*=O), 167.58 (2C, (*C*=O)OEt), 137.64 (2C, Ar *C*), 135.05 (Ar *C*), 128.80 (Ar *C*), 127.75 (2C, Ar *C*), 67.26 (quaternary C), 62.57 (2C, CH_2CH_3), 37.60 (Ar-CH_2), 22.99 ((C=O)CH_3), 21.23 (2C, Ar-CH_3), 14.04 (2C, CH_2CH_3). MS (ESI): *m/z* (%): 358.4 ($[M + Na]^+$) (100), 336.5 ($[M + H]^+$) (10). Anal. calcd for $C_{18}H_{25}NO_5$: C 64.46, H 7.51, N 4.18; found: C 64.57, H 7.70, N 4.31.

3,5-Dimethyl-DL-phenylalanine

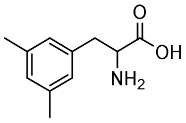

3,5-Dimethyl-DL-phenylalanine was prepared from diethyl 2-acetamido-2-(3,5-dimethylbenzyl)malonate by the method of Dailey.[1] A suspension of diethyl 2-acetamido-2-(3,5-dimethylbenzyl)malonate (0.5 g, 0.0015 mol) in 37% aqueous hydrochloric acid (6.0 mL) was heated under reflux for eight hours and then filtered. The filtrate was diluted with water (25.0 mL), concentrated and cooled in an ice-bath, giving 3,5-dimethyl-DL-phenylalanine as colourless crystals. Yield 0.81 g, 87%. mp 224.1−231.1 °C decomp. (lit. mp 229−231 °C[1] and 212−214 °C[6]). ^{1}H NMR (400 MHz, D_2O): δ 6.98 (s, 1H; Ar H), 6.92 (s, 2H; Ar H), 3.45−3.48 (m, 1H; C*H*), 2.91−2.92 (m, 2H; C*H*HCH), 2.73-2.77 (m, 2H; CH*H*CH), 2.28-2.29 ppm (m, 6H; $(CH_3)_2$). ^{13}C NMR (101 MHz, [D6]DMSO): δ 170.58 (*C*=O), 137.88 (2C, Ar *C*), 135.02 (Ar *C*), 129.05 (Ar *C*), 127.63 (2C, Ar *C*), 53.64 (*C*H), 35.91 (CH_2), 21.33 ppm (2C, CH_3). MS (ESI): *m/z* (%): 216.5 ($[M + Na]^+$) (100), 194.4 ($[M + H]^+$) (40). Anal. calcd for $C_{11}H_{16}NO_2Cl$: C 57.52, H 7.02, N 6.10; found: C 57.48, H 7.10, N 6.01. The spectral properties of this compound were identical with those previously reported for

3,5-dimethyl-DL-phenylalanine.[6]

Diethyl 2-acetamido-2-(3,5-dimethoxybenzyl)malonate

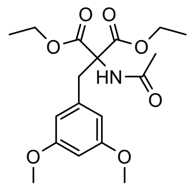

Diethyl 2-acetamido-2-(3,5-dimethoxybenzyl)malonate was prepared from 1-(bromomethyl)-3,5-dimethoxybenzene by the method of Warne.[5] To a solution of metallic sodium (0.624 g, 0.0271 mol) in dry ethanol (40.0 mL), diethyl acetaminomalonate (2.82 g, 0.013 mol) was added. The resultant mixture was stirred at room temperature for 30 minutes, mixed with 1-(bromomethyl)-3,5-dimethoxybenzene (3.0 g, 0.013 mol), heated under reflux for 1.5 hours, concentrated and extracted with ethyl acetate (3 × 15 mL). The combined acetate extracts were washed with saturated brine, dried (sodium sulfate), filtered and concentrated, yielding a residue which was recrystallized from ethyl acetate and hexane to give diethyl 2-acetamido-2-(3,5-dimethoxybenzyl)malonate as a white solid. Yield 1.138 g, 81%. mp 113.3−114.9 °C (lit.[5] mp 114 °C); ^{1}H NMR (400 MHz, $CDCl_3$): δ 6.59 (s, 1H; N*H*), 6.32−6.13 (m, 3H; Ar H), 4.27−4.24 (m, 4H; ($CH_2CH_3)_2$), 3.71 (s, 6H; ($OCH_3)_2$), 3.56 (s, 2H; Ar-C*H*$_2$), 2.02 (s, 3H; (C=O)C*H*$_3$), 1.28 (t, J = 7.2 Hz, 6H, ($CH_2CH_3)_2$). ^{13}C NMR (101 MHz, $CDCl_3$): δ 169.09 (NH*C*=O), 167.56 (2C, (*C*=O)OEt), 160.68 (2C, Ar *C*), 137.51 (Ar *C*), 108.10 (2C, Ar *C*), 99.07 (Ar *C*), 67.23 (quaternary C), 62.74 (2C, *C*H$_2$CH$_3$), 55.26 (2C, O*C*H$_3$), 38.10 (Ar-*C*H$_2$), 23.13 ((C=O)*C*H$_3$), 14.10 (2C, CH$_2$*C*H$_3$). MS (ESI): *m*/*z* (%): 390.5 ([M + Na]$^+$) (100), 368.6 ([M + H]$^+$) (20). Anal. calcd for $C_{18}H_{25}NO_7$: C 58.85, H 6.86, N 3.81; found: C 58.85, H 6.99, N 3.91. The spectral properties of this compound were identical with those previously reported for Diethyl 2-acetamido-2-(3,5-dimethoxybenzyl)malonate.[7]

3,5-Dimethoxy-DL-phenylalanine

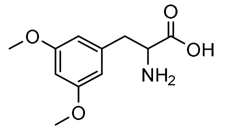

3,5-Dimethoxy-DL-phenylalanine was prepared from diethyl 2-acetamido-2-(3,5-dimethoxybenzyl)malonate by the method of Warne.[5] A solution of diethyl 2-acetamido-2-(3,5-dimethoxybenzyl)malonate (1.0 g, 0.0027 mol) in 37% aqueous hydrochloric acid (10.0 mL) and acetic acid (10.0 mL) was heated under reflux for 1.5 hours and then concentrated. Recrystallization of the residue from ethanol gave 3,5-dimethoxy-DL-phenylalanine as a white solid. Yield 0.57 g, 93%. mp 227.8−231.2 °C. ^{1}H NMR (400 MHz, CD_3OD): δ 6.35−6.36 (m, 2H; Ar H), 6.30-6.31 (m, 1H; Ar H), 4.15 (dd, J = 7.6, 5.2 Hz, 1H; C*H*), 3.65 (s, 6H; ($CH_3)_2$), 3.13 (dd, J = 14.4, 5.2 Hz, 1H; C*H*HCH), 2.98 (dd, J = 14.4, 7.6 Hz, 1H; CH*H*CH). ^{13}C NMR (101 MHz, CD_3OD): δ 171.19 (*C*=O), 162.81 (2C, Ar *C*), 137.54 (Ar *C*), 108.38 (2C, Ar *C*), 100.65 (Ar *C*), 55.80 (2C, *C*H$_3$), 54.95 (*C*H), 37.43 (*C*H$_2$). MS (ESI): *m*/*z* (%): 248.6 ([M + Na]$^+$) (100), 226.6 ([M + H]$^+$) (5). Anal. calcd for $C_{11}H_{16}NO_4Cl$: C 50.48, H 6.16, N 5.35; found: C 50.45, H 6.25, N 5.30.

3,5-Dihydroxy-DL-phenylalanine

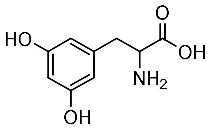

3,5-Dihydroxy-DL-phenylalanine was prepared from (*R,S*)-2-amino-3-(3,5-dimethoxyphenyl)propanoic acid by the method reported previously.[7,8] A solution of (*R,S*)-2-amino-3-(3,5-dimethoxyphenyl)propanoic acid (0.5 g, 0.0022 mol) in 62% aqueous hydrobromic acid (11.5 mL) and acetic acid (7.0 mL) was heated in a sealed tube at 150 °C for two hours. The mixture was diluted with water (10.0 mL), decolorized with charcoal and concentrated, affording a light brown residue which was recrystallized from ethanol to gave 3,5-dihydroxy-DL-phenylalanine as a white solid. Yield 0.28 g, 63%. Mp 307.4−309.6 °C decomp. (lit.[8] mp 312 °C decomp.). ^{1}H NMR (400 MHz, D_2O): δ=6.32−6.31 (m, 3H; Ar H), 4.28 (dd, *J* = 7.6, 5.6 Hz, 1H; C*H*), 3.20 (dd, *J* = 14.4, 5.6 Hz, 1H; C*H*HCH), 3.01 (dd, *J* = 14.4, 7.6 Hz, 1H; CH*H*CH). ^{13}C NMR (101 MHz, D_2O): δ 171.18 (*C*=O), 157.18 (2C, Ar *C*), 136.72 (Ar *C*), 108.43 (2C, Ar *C*), 101.98 (Ar *C*), 53.73 (*C*H), 35.26 ppm (*C*H_2). MS (ESI): *m*/*z* (%): 220.2 ([M + Na]$^+$) (100), 198.4 ([M + H]$^+$) (50). Anal. calcd for $C_9H_{11}NO_4$: C 54.82, H 5.62, N 7.10; found: C 54.47, H 6.02, N 6.98.

1-(Bromomethyl)-3-methoxy-5-methylbenzene

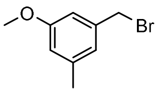

1-(Bromomethyl)-3-methoxy-5-methylbenzene was prepared from 1-methoxy-3,5-dimethylbenzene by the method of Bickelhaupt.[9] The choice of solvent for bromination was based on the work of Golding.[2] To a solution of 1-methoxy-3,5-dimethylbenzene (2.0 g, 0.015 mol) and *N*-bromosuccinimide (2.655 g, 0.015 mol) in α,α,α-trifluorotoluene (160.0 mL) and water (1.32 g, 0.073 mol), benzoyl peroxide (0.363 g, 0.01 mol) was added. The resultant mixture was heated under reflux for one hour, then filtered. The filtrate was washed with 3.0 M aqueous hydrochloric acid (3 × 50 mL), saturated sodium bicarbonate (3 × 50 mL), water (3 × 50 mL), brine (3 × 50 mL), and then dried (sodium sulphate) and concentrated, yielding a residue which, upon purification with chromatography from dichloromethane and hexane, gave 1-(bromomethyl)-3-methoxy-5-methylbenzene as a semi-solid at room temperature (lit.[10] mp 38 °C). Yield 2.64 g, 82%. ^{1}H NMR (400 MHz, $CDCl_3$): δ 6.68−6.57 (m, 3H; Ar H), 4.46 (s, 2H; C*H*$_2$), 3.82 (s, 3H; OC*H*$_3$), 2.42 (s, 3H; Ar-C*H*$_3$). ^{13}C NMR (101 MHz, $CDCl_3$): δ 160.12 (Ar *C*), 141.64 (Ar *C*), 138.98 (Ar *C*), 123.28 (Ar *C*), 113.55 (Ar *C*), 111.62 (Ar *C*), 56.01 (O*C*H_3), 34.23 (*C*H_2), 22.18 (Ar-*C*H_3). MS (ESI): *m*/*z* (%): 135.0 ($C_9H_{11}O$) (100), 216.3 ([M + H]$^+$) (40). The spectral properties of this compound were identical with those previously reported for 1-(bromomethyl)-3-methoxy-5-methylbenzene.[10,11]

2-Fluoro-4-methoxybenzoyl chloride

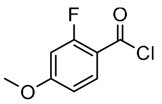

2-Fluoro-4-methoxybenzoyl chloride was prepared from 2-fluoro-4-methoxybenzoic acid by the method of Bennett and Niemann.[12] A solution of 2-fluoro-4-methoxybenzoic acid (5.0 g, 0.029 mol) in thionyl chloride (7.0 mL) was heated under reflux overnight. The mixture was concentrated, and the resultant residue was recrystallized from petroleum to give 2-fluoro-4-methoxybenzoyl chloride as colourless crystals. Yield 5.25g, 95%. Mp 39.9−41.2 °C (lit.[12] mp 37−40 °C). ^{1}H NMR (400 MHz, $CDCl_3$): δ 8.03 (triplet, *J* = 8.8 Hz, 1H; Ar H), 6.75 (dd, *J* = 8.8, 2.4 Hz, 1H; Ar H), 6.60-6.6 (m, 1H; Ar H), 3.88 (s, 3H; C*H*$_3$). ^{13}C NMR (100 MHz, $CDCl_3$): δ 166.73 (d, *J* = 12.1 Hz, Ar *C*),

161.81-162.25 (2C, *C*=O and Ar *C*), 136.34 (Ar *C*), 113.72 (d, *J* = 7.1 Hz, Ar *C*), 110.72 (Ar *C*), 102.48 (d, *J* = 24.5 Hz, Ar *C*), 56.25 (CH_3). MS (ESI): *m*/*z* (%) 211.71 ([M + Na]$^+$) (100), 189.62 ([M + H]$^+$) (20);

(2-Fluoro-4-methoxyphenyl)methanol

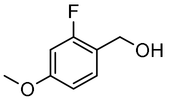

(2-Fluoro-4-methoxyphenyl)methanol was prepared from 2-fluoro-4-methoxybenzoyl chloride by the method of Bennett and Niemann.[12] To an ice-cooled solution of 2-fluoro-4-methoxybenzoyl chloride (5.0 g, 0.0266 mol) in anhydrous diethyl ether (20.0 mL), 0.5 M lithium aluminium hydride in dry diethyl ether was added dropwise while the temperature was kept at 0 °C. The mixture was stirred at room temperature for 20 minutes. Then water (5.0 mL) was added dropwise. The resultant mixture was poured onto ice water in 5% sulfuric acid. The ether phase was collected and the aqueous phase was extracted with diethyl ether (2 × 10 mL). The combined ether extracts were washed with water until neutral, dried (sodium sulphate) and concentrated, giving the crude product mixture as light green liquid. It was shown by ^{1}H NMR to be the major component of the crude product mixture. The crude product mixture was used on the next reaction without further purification, as described in literature.[12]

1-(Chloromethyl)-2-fluoro-4-methoxybenzene

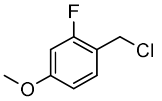

1-(Chloromethyl)-2-fluoro-4-methoxybenzene was prepared from (2-fluoro-4-methoxyphenyl)methanol by the method of Bennett and Niemann.[12] A solution of the crude product mixture of (2-fluoro-4-methoxyphenyl)methanol in thionyl chloride (25.0 mL) was stirred at room temperature overnight and then warmed at 95 °C for one hour. The mixture was concentrated and purified using chromatography from petroleum to give 1-(chloromethyl)-2-fluoro-4-methoxybenzene as a clear liquid. The total yield of the reactions over two steps, from 2-fluoro-4-methoxybenzoyl chloride to (2-fluoro-4-methoxyphenyl)methanol, and to 1-(chloromethyl)-2-fluoro-4-methoxybenzene is 3.7 g, 80%. ^{1}H NMR (400 MHz, $CDCl_3$): δ 7.29 (triplet, *J* = 8.8 Hz, 1H; Ar H), 6.69 (dd, *J* = 8.8, 2.8 Hz, 1H; Ar H), 6.62-6.65 (m, 1H; Ar H), 4.61 (s, 2H; CH_2), 3.79 (s, 3H; CH_3). ^{13}C NMR (100 MHz, $CDCl_3$): δ 162.65 (Ar *C*), 161.48 (d, *J* = 11.0 Hz, Ar *C*), 131.59 (d, *J* = 5.0 Hz, Ar *C*), 116.86 (d, *J* = 15.0 Hz, Ar *C*), 110.32 (Ar *C*), 101.84 (d, *J* =24.8 Hz, Ar *C*), 55.67 (CH_3), 39.69 (CH_2). MS (ESI): *m*/*z* (%) 197.7 ([M + Na]$^+$) (100), 175.8 ([M + H]$^+$) (80). Anal. calcd for C_8H_8ClFO: C 55.03, H 4.62; found: C 54.96, H 4.61.

Diethyl 2-acetamido-2-(2-fluoro-4-methoxybenzyl)malonate

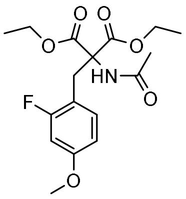

Diethyl 2-acetamido-2-(2-fluoro-4-methoxybenzyl)malonate was prepared from 1-(chloromethyl)-2-fluoro-4-methoxybenzene by the method of Bennett and Niemann.[12] To a solution of metallic sodium (0.51 g, 0.022 mol) in dry ethanol (40.0 mL), diethyl acetaminomalonate (4.78 g, 0.022 mol) was added. The

resultant mixture was stirred at room temperature for 30 minutes, mixed with 1-(chloromethyl)-2-fluoro-4-methoxybenzene (3.5 g, 0.02 mol), heated under reflux for four hours, concentrated and extracted with ethyl acetate (3 × 10 mL). The combined extracts were washed with saturated brine, dried (sodium sulfate), filtered and concentrated, yielding a residue which was recrystallized from ethyl acetate and hexane to give diethyl 2-acetamido-2-(2-fluoro-4-methoxybenzyl)malonate as a white solid. Yield 7.83 g, 69%. mp 117.3−119.4 °C (lit. mp 120−121.5 °C[12] and 122−124 °C[13]). ^{1}H NMR (400 MHz, $CDCl_3$): δ 6.88 (t, J = 8.8 Hz, 1H; Ar H), 6.57 (dd, *J* = 8.8, 2.8 Hz, 1H; Ar H), 6.49-6.53 (m, 1H; Ar H), 4.23 (q, *J* = 7.2 Hz, 4H; $(OCH_2CH_3)_2$), 3.73 (s, 3H; OCH_3), 3.60 (s, 2H; Ar-CH_2), 1.96 (s, 3H; (C=O)CH_3), 1.25 (t, *J* = 7.2 Hz, 6H; $(OCH_2CH_3)_2$). ^{13}C NMR (100 MHz, $CDCl_3$): δ 169.20 (NH*C*=O), 167.67 (2C, (*C*=O)OEt), 163.22 (Ar *C*), 160.17 (d, *J* = 11.0 Hz, Ar *C*), 132.68 (d, *J* = 6.6 Hz, Ar *C*), 113.78 (d, *J* = 16.6 Hz, Ar *C*), 109.95 (Ar *C*), 101.42 (d, *J* = 26.2 Hz, Ar *C*), 66.34 (quaternary C), 62.68(2C, OCH_2), 55.49 (OCH_3), 31.30 (Ar−CH_2), 22.90 ((C=O)CH_3), 13.96 ppm (2C, CH_3). MS (ESI): *m*/*z* (%): 378.5 ([M + Na]$^+$) (100), 356.6 ([M + H]$^+$) (20). Anal. calcd for $C_{17}H_{22}O_6NF$: C 57.46, H 6.24, N 3.94; found: C 57.47, H 6.11, N 3.88.

2-Acetamido-3-(2-fluoro-4-methoxyphenyl)propanoic acid

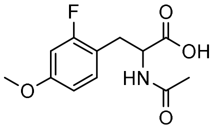

2-Acetamido-3-(2-fluoro-4-methoxyphenyl)propanoic acid was prepared from diethyl 2-acetamido-2-(2-fluoro-4-methoxybenzyl)malonate by the method of Bennett and Niemann.[121] A suspension of diethyl 2-acetamido-2-(2-fluoro-4-methoxybenzyl)malonate (2.0 g, 0.0056 mol) in 2.5 M aqueous sodium hydroxide (10.0 mL) was heated under reflux for six hours. Then 5.0 M aqueous hydrochloric acid (10.0 mL) was added and the mixture was heated under reflux for additional two hours, affording, after standing at 4 °C overnight, precipitate which was recrystallized from ethanol to give 2-acetamido-3-(2-fluoro-4-methoxyphenyl)propanoic acid as colourless crystals. Yield 1.15 g, 80%. mp 168.2−171.3 °C (lit.[12] mp 169.5−171.5 °C). ^{1}H NMR (400 MHz, D_2O): δ 7.08-7.13 (m, 1H; Ar H), 6.61-6.66 (m, 2H; Ar H), 4.38 (dd, *J* = 9.2, 5.2 Hz, 1H; CH_2CH), 3.70 (s, 3H; OCH_3), 3.14 (dd, *J* = 14.0, 5.2 Hz, 1H; C*H*HCH), 2.75-2.81 (dd, *J* = 14.0, 9.2 Hz, 1H; C*H*HCH), 1.87 (s, 3H; (C=O)CH_3). ^{13}C NMR (100 MHz, D_2O): δ 177.08 ((*C*=O)OH), 172.24 (NH*C*=O), 161.88 (Ar *C*), 158.05 (d, *J* = 11.1 Hz, Ar *C*), 131.02 (Ar *C*), 115.77 (d, *J* = 16.2 Hz, Ar *C*), 108.96 (Ar *C*), 100.64 (d, *J* = 26.1 Hz, Ar *C*), 54.65 (*C*H), 54.62 (OCH_3), 29.94 (CH_2), 20.99 ((*C*=O)CH_3). MS (ESI): *m*/*z* (%): 236.4 ([M + Na]$^+$) (100), 214.6 ([M + H]$^+$) (60). Anal. calcd for $C_{12}H_{14}O_4NF$: C 56.47, H 5.53, N 5.49; found: C 56.26, H 5.58, N 5.34.

***o*-Fluoro-DL-tyrosine**

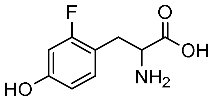

o-Fluoro-DL-tyrosine was prepared from 2-acetamido-3-(2-fluoro-4-methoxyphenyl)propanoic acid by the method used for the preparation of (*R*,*S*)-2-amino-3-(3,5-dimethoxyphenyl)propanoic acid from (*R*,*S*)-3,5-dihydroxyphenylalanine.[5] A solution of 2-acetamido-3-(2-fluoro-4-methoxyphenyl)propanoic acid (1.0 g, 0.0039 mol) in 62% aqueous hydrobromic acid (11.5 mL) and acetic acid (7.0 mL) was heated in a sealed tube at 150 °C for two hours. The mixture was diluted with water (10.0 mL), decolorized with charcoal and concentrated, affording a residue which was recrystallized from water and ethanol to give *o*-fluoro-DL-tyrosine as a white solid. Yield 0.5 g, 64%. mp

265.9−270.4 °C decomp. (lit. mp 280−285 °C decomp.[12] and 293−294 °C decomp.[13]). ^{1}H NMR (400 MHz, D_2O): δ 6.96-7.01 (m, 1H; Ar H), 6.35-6.44 (m, 2H; Ar H), 3.51-3.55 (m, 1H; CH_2CH), 2.94-2.99 (m, 1H; C*H*HCH), 2.74-2.79 (m, 1H; CH*H*CH). ^{13}C NMR (100 MHz, $CDCl_3$): δ 180.87 ((*C*=O)OH), 165.59 (d, *J* = 11.3 Hz, Ar *C*), 160.95 (Ar *C*), 131.66 (d, *J* = 7.8 Hz, Ar *C*), 114.33 (Ar *C*), 109.98 (d, *J* = 15.6 Hz, Ar *C*), 104.46 (d, *J* = 19.9 Hz, Ar *C*), 56.56 (*C*H), 32.75 ppm (*C*H_2). MS (ESI): *m*/*z* (%): 222.4 ([M + Na]$^+$) (100), 200.5 ([M + H]$^+$) (75). Anal. calcd for $C_9H_{10}O_3NF$: C 54.27, H 5.06, N 7.03; found: C 53.89, H 5.22, N 6.83. The spectral properties of this compound were identical with those previously reported for *o*-fluoro-DL-tyrosine.[14]

2,4-Dimethoxybenzaldehyde

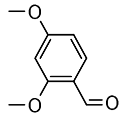

2,4-Dimethoxybenzaldehyde was prepared from 2,4-dihydroxybenzaldehyde by the method of Vaya.[15] To a solution of 2,4-dihydroxybenzaldehyde (2.0 g, 0.0145 mol) and cesium carbonate (14.1 g, 0.0435 mol) in dry dimethylformamide (200.0 mL), methyl iodide (12.4 g, 0.087 mol) was added dropwise. The resultant mixture was heated under reflux for two hours, then cooled and filtered. The filtrate was concentrated, redissolved in dichloromethane (60.0 mL), washed with water (3 × 20 mL), dried (sodium sulphate) and concentrated to yield a residue which was purified with chromatography from ethyl acetate and hexane to give 2,4-dimethoxybenzaldehyde as a white solid. Yield 2.29g, 95%. mp 70.2−70.8 °C (lit.[8] mp 70−71 °C). ^{1}H NMR (400 MHz, $CDCl_3$): δ=10.13 (s, 1H; (C=O)*H*), 7.58 (d, *J* = 12.0 Hz, 1H, Ar H), 6.51 (dd, *J* = 12.0, 2.8 Hz, 1H, Ar H), 6.47 (d, *J* = 2.8 Hz, 1H, Ar H), 3.82 (s, 3H; OCH_3), 3.77 (s, 3H; OCH_3). ^{13}C NMR (100 MHz, $CDCl_3$): δ 205.33 (*C*=O)H), 166.34 (Ar *C*), 163.31 (Ar *C*), 129.48 (Ar *C*), 118.75 (Ar *C*), 106.40 (Ar *C*), 97.75 (Ar *C*), 55.35 (2C, O*C*H_3). MS (ESI): *m*/*z* (%): 189.3 ([M + Na]$^+$) (100), 167.5 ([M + H]$^+$) (30). The spectral properties of this compound were identical with those previously reported for 2,4-dimethoxybenzaldehyde.[16]

(*Z*)-4-(2,4-dimethoxybenzylidene)-2-phenyloxazol-5(4H)-one

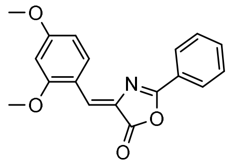

(*Z*)-4-(2,4-dimethoxybenzylidene)-2-phenyloxazol-5(4H)-one was prepared from 2,4-dimethoxybenzaldehyde by the method of Lambooy.[116] A suspension of 2,4-dimethoxybenzaldehyde (2.0 g, 0.012 mol), benzoylaminoethanoic acid (2.59 g, 0.014 mol) and sodium acetate (1.48 g, 0.018 mol) in dry acetic anhydride (10.0 mL) was heated under reflux for one hour, then concentrated to yield a yellow residue which was suspended in water (10.0 mL) and permitted to stand overnight. The precipitate was recrystallized from ethanol to give (*Z*)-4-(2,4-dimethoxybenzylidene)-2-phenyloxazol-5(4*H*)-one as a yellow solid. Yield 3.23 g, 87%. mp 163.1−180.9 °C (lit.[8] mp 173−178 °C). ^{1}H NMR (400 MHz, CD_3OD): δ 7.91 (d, *J* = 11.2 Hz, 1H; (C=O)*H*), 7.82 (m, 2H; Ar H), 7.64-7.67 (m, 1H; Ar H), 7.53-7.56 (m, 1H; Ar H), 7.44-7.47 (m, 2H; Ar H), 6.53 (dd, *J* = 11.2, 3.2 Hz, 1H, Ar H), 6.43 (d, *J* = 3.2 Hz, 1H, Ar H), 3.82 (s, 3H; OCH_3), 3.73 (s, 3H; OCH_3). ^{13}C NMR (100 MHz, CD_3OD): δ=168.27 (*C*=O), 167.36 (Ar-*C*=N), 162.71 (Ar *C*), 159.52 (Ar *C*), 133.69 (*C*(C=O)N=C), 131.71 (Ar *C*), 130.08 (Ar *C*), 128.26 (2C, Ar *C*), 127.33 (2C, Ar *C*), 123.04 (Ar *C*), 114.99 (*C*H), 105.02 (2C, Ar *C*), 97.64 (Ar *C*), 54.88 (O*C*H_3), 53.51

(OCH_3). MS (ESI): *m*/*z* (%): 332.6 ([M + Na]$^+$) (100), 310.5 ([M + H]$^+$) (40). The spectral properties of this compound were identical with those previously reported for (*Z*)-4-(2,4-dimethoxybenzylidene)-2-phenyloxazol-5(4*H*)-one.[17]

(*Z*)-2-benzamido-3-(2,4-dimethoxyphenyl)acrylic acid

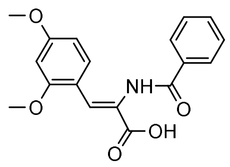

(*Z*)-2-benzamido-3-(2,4-dimethoxyphenyl)acrylic acid was prepared from (*Z*)-4-(2,4-dimethoxybenzylidene)-2-phenyloxazol-5(4*H*)-one by the method of Lambooy.[8] To a solution of (*Z*)-4-(2,4-dimethoxybenzylidene)-2-phenyloxazol-5(4*H*)-one (2.0 g, 0.0065 mol) in ethanol (30.0 mL) that had been heated to 90 °C, 0.5 M aqueous sodium hydroxide (17.0 mL) was added dropwise. The mixture was held at 90 °C for two hours, then cooled, acidified with 0.5 M aqueous hydrochloric acid and kept at 0 °C overnight, affording precipitate which was recrystallized from water and ethanol to give (*Z*)-2-benzamido-3-(2,4-dimethoxyphenyl)acrylic acid as colourless needles. Yield 1.99 g, 94%. mp 210.3−213.4 °C decomp. (lit.[8] mp 229−230 °C). ^{1}H NMR (400 MHz, CD_3OD): δ=7.91-7.93 (m, 3H; (C=O)*H*, Ar H), 7.63 (7.68 (d, *J* = 8.4 Hz, 1H; Ar H), 7.55-7.58 (m, 1H; Ar H), 7.47-7.50 (m, 2H; Ar H), 6.54 (d, *J* = 2.0 Hz, 1H; Ar H), 6.47 (dd, *J* = 8.8, 2.0 Hz, 1H; Ar H), 3.86 (s, 3H; OCH_3), 3.77 (s, 3H; OCH_3). ^{13}C NMR (100 MHz, CD_3OD): δ 169.71(Ar-*C*=O), 168.75 ((*C*=O)OH), 164.10 (Ar *C*), 160.91 (Ar *C*), 135 (Ar *C*), 133.10 (Ar *C*), 131.17 (Ar *C*), 129.65 (2C, Ar *C*), 128.72 (2C, Ar *C*), 124.43 (*C*H), 116.39 (*C*(C=O)NH), 106.41 (2C, Ar *C*), 99.04 (Ar *C*), 56.27 (OCH_3), 55.90 (OCH_3). MS (ESI): *m*/*z* (%): 350.6 ([M + Na]$^+$) (100), 328.4 ([M + H]$^+$) (70);

2-Benzamido-3-(2,4-dimethoxyphenyl)propanoic acid

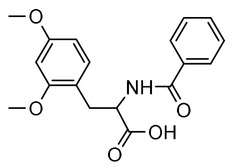

2-Benzamido-3-(2,4-dimethoxyphenyl)propanoic acid was prepared from (*Z*)-2-benzamido-3-(2,4-dimethoxyphenyl)acrylic acid by the method of Lambooy.[8] A solution of (*Z*)-2-benzamido-3-(2,4-dimethoxyphenyl)acrylic acid (1.5 g, 0.0046 mol) in 0.3 M aqueous sodium hydroxide (25.0 mL) with palladium on carbon (0.075 g) was hydrogenated at 60 p.s.i. at room temperature for two hours. The mixture was filtered through celite, acidified and recrystallized from water and acetic acid to give 2-benzamido-3-(2,4-dimethoxyphenyl)propanoic acid colourless needles. Yield 1.33 g, 88%. mp 150.3−164.2 °C (lit.[8] mp 169 °C). ^{1}H NMR (400 MHz, CD_3OD): δ 7.70 (m, 2H; Ar H), 7.49−7.40 (m, 3H; Ar H), 7.11 (d, *J* = 8.4 Hz, 1H; Ar H), 6.47 (d, *J* = 2.4 Hz, 1H; Ar H), 6.41 (dd, *J* = 8.4, 2.4 Hz, 1H; Ar H), 4.71 (m, 1H; C*H*), 3.75 (s, 3H; CH_3), 3.71 (s, 3H; CH_3), 3.32 (m, 1H; C*H*HCH), 3.01 (m, 1H; CH*H*CH). ^{13}C NMR (100 MHz, CD_3OD): δ 177.09 ((*C*=O)OH), 168.34 (Ar-*C*=O), 160.02 (Ar-*C*), 158.47 (Ar-*C*), 134.23 (Ar-*C*), 131.25 (Ar-*C*), 131.10 (Ar-*C*), 128.11(2C; Ar-*C*), 126.76 (2C; Ar-*C*), 118.17 (Ar-*C*), 109.98 (Ar-*C*), 97.82 (Ar-*C*), 55.51 (*C*H), 54.47 (2C; *C*H_3), 31.56 (*C*H_2). MS (ESI): *m*/*z* (%): 352.5 ([M + Na]$^+$) (100), 330.6 ([M + H]$^+$) (60).

2,4-Dihydroxy-DL-phenylalanine

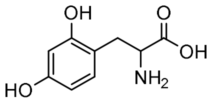

2,4-Dihydroxy-DL-phenylalanine was prepared from 2-benzamido-3-(2,4-dimethoxyphenyl)propanoic acid by the method of Lambooy.[8] A solution of 2-benzamido-3-(2,4-dimethoxyphenyl)propanoic acid (1.0 g, 0.003 mol) in 62% aqueous hydrobromic acid (10.0 mL) and acetic acid (5.0 mL) was heated in a sealed tube under nitrogen at 150 °C−160 °C for two hours. The mixture was diluted with water (10.0 mL), decolorized with charcoal and concentrated, affording a residue which recrystallized from water to give 2,4-dihydroxy-DL-phenylalanine as white solid. Yield 0.41 g, 68%. mp 247.3−252.1 °C decomp. (lit.[8] mp 255−257 °C decomp.). ^{1}H NMR (400 MHz, CD_3OD): δ=6.92-6.93 (m, 1H; Ar H), 6.27−6.38 (m, 2H; Ar H), 4.20-4.22 (m, 1H; C*H*), 3.27-3.28 (m, 1H; C*H*HCH), 2.92-2.97 (m, 1H; CH*H*CH). ^{13}C NMR (100 MHz, CD_3OD): δ 171.65 (*C*=O), 159.29 (Ar *C*), 157.77 (Ar *C*), 132.94 (Ar *C*), 112.99 (Ar *C*), 108.51 (Ar *C*), 103.66 (Ar *C*), 54.56 (*C*H), 32.28 ppm (*C*H_2). MS (ESI): *m*/*z* (%): 220.4 ($[M + Na]^+$) (100), 198.6 ($[M + H]^+$) (20). Anal. calcd for $C_9H_{11}NO_4$: C 54.82, H 5.62, N 7.10; found: C 55.01, H 5.90, N 6.91.

Diethyl 2-acetamido-2-(2-chloro-4-methoxybenzyl)malonate

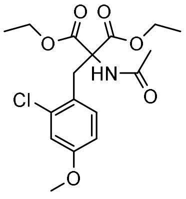

Diethyl 2-acetamido-2-(2-chloro-4-methoxybenzyl)malonate was prepared from 1-(bromomethyl)-2-chloro-4-methoxybenzene by the method of McCord.[13] To a solution of metallic sodium (0.11 g, 0.0047 mol) in dry ethanol (10.0 mL), diethyl acetaminomalonate (1.01 g, 0.0047 mol) was added. The resultant mixture was stirred at room temperature for 30 minutes, mixed with 1-(bromomethyl)-2-chloro-4-methoxybenzene (1.0 g, 0.0042 mol), stirred at room temperature for three hours, concentrated and extracted with ethyl acetate (3 × 4 mL). The combined extracts were washed with saturated brine, dried (sodium sulfate), filtered and concentrated, yielding a residue which was recrystallized from ethyl acetate and hexane to give diethyl 2-acetamido-2-(2-chloro-4-methoxybenzyl)malonate as white solid. Yield 1.45 g, 92%. mp 129−131.4 °C (lit.[13] mp 128−129 °C). ^{1}H NMR (400 MHz, $CDCl_3$): δ 6.95 (d, *J* = 8.4 Hz, 1H; Ar H), 6.85 (d, *J* = 2.4 Hz, 1H; Ar H), 6.71 (dd, *J* = 8.4, 2.4 Hz, 1H; Ar H), 6.51 (s, 1H; N*H*), 4.24 (q, *J* = 7.2 Hz, 4H; (OC*H*$_2$$CH_3$)$_2$), 3.75 (s, 3H; OC*H*$_3$), 3.72 (s, 2H; Ar-C*H*$_2$), 1.99 (s, 3H; (C=O)C*H*$_3$), 1.29 (t, *J* = 7.2 Hz, 6H; (OCH_2C*H*$_3$)$_2$). ^{13}C NMR (100 MHz, $CDCl_3$): δ 169.36 (NH*C*=O), 167.79 (2C, (*C*=O)OEt), 159.31 (Ar *C*), 135.46 (Ar *C*), 132.75 (Ar *C*), 125.05 (Ar *C*), 114.84 (Ar *C*), 113.03 (Ar *C*), 66.39 (quaternary C), 62.74 (2C, *C*$H_2$$CH_3$), 55.53 (O*C*$H_3$), 34.46 (Ar-*C*$H_2$), 23.13 ((C=O)*C*$H_3$), 14.02 (2C, O$CH_2$*C*$H_3$). MS (ESI): *m*/*z* (%): 395.0 ($[M + Na]^+$) (100), 372.2 ($[M + H]^+$) (50). Anal. calcd for $C_{17}H_{22}ClNO_6$: C 54.92, H 5.96, N 3.77; found: C 54.90, H 5.93, N 3.77.

***o*-Chloro-DL-tyrosine**

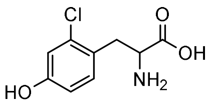

o-Chloro-DL-tyrosine was prepared from diethyl 2-acetamido-2-(2-chloro-4-methoxybenzyl)malonate by the method of McCord.[13] A solution of diethyl 2-acetamido-2-(2-chloro-4-methoxybenzyl)malonate (1.0 g, 0.0027 mol) in 62%

aqueous hydrobromic acid (5.5 mL) and acetic acid (3.0 mL) was heated in a sealed tube at 150 °C for three hours. The mixture was diluted with water (10.0 mL), decolorized with charcoal and concentrated, affording a residue which was recrystallized from water and ethanol to give *o*-chloro-DL-tyrosine as white solid. Yield 0.39 g, 67%. mp 251.2−254.7 °C decomp. (lit.[13] mp 256−257 °C decomp.). ^{1}H NMR (400 MHz, CD_3OD): δ 6.85 (d, *J* = 8.4 Hz, 1H; Ar H), 6.56 (d, *J* = 2.4 Hz, 1H; Ar H), 6.42 (dd, *J* = 8.4, 2.4 Hz, 1H; Ar H), 3.86 (dd, *J* = 8.4, 6.4 Hz, 1H; CH_2CH), 3.07 (dd, *J* = 14.4, 6.4 Hz, 1H; C*H*HCH), 2.79 (dd, *J* = 14.4, 8.4 Hz, 1H; CH*H*CH). ^{13}C NMR (100 MHz, CD_3OD): δ 172.06 (*C*=O), 160.24 (Ar *C*), 136.61 (Ar *C*), 134.43 (Ar *C*), 124.63 (Ar *C*), 118.53 (Ar *C*), 116.69 (Ar *C*), 54.82 (*C*H), 35.62 ppm (CH_2). MS (ESI): *m*/*z* (%): 238.8 ($[M + Na]^+$) (100), 216.9 ($[M + H]^+$) (75). Anal. calcd for $C_9H_{10}ClNO_3$: C 50.13, H 4.67, N 6.5; found: C 50.17, H 4.64, N 6.44. The spectral properties of this compound were identical with those previously reported for *o*-chloro-DL-tyrosine.[14]

Diethyl 2-acetamido-2-(4-methoxy-3-methylbenzyl)malonate

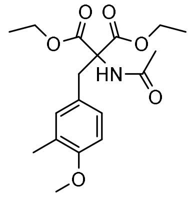

Diethyl 2-acetamido-2-(4-methoxy-3-methylbenzyl)malonate was prepared from 4-(chloromethyl)-1-methoxy-2-methylbenzene by the method of Wiley.[18] A solution of metallic sodium (0.15 g, 0.0065 mol) and diethyl acetaminomalonate (1.4 g, 0.0065 mol) in dry ethanol (10.0 mL) was stirred at room temperature for 30 minutes. To the resultant mixture, 4-(chloromethyl)-1-methoxy-2-methylbenzene (1.0 g, 0.0059 mol) was added dropwise. The mixture was heated at reflux for 1.5 hours, then poured onto ice water. The residue oil, which solidified soon after separation, was recrystallized from henxane to give diethyl 2-acetamido-2-(4-methoxy-3-methylbenzyl)malonate as colourless crystals. Yield 1.51 g, 73%. mp 110.3−111.9 °C (lit.[18] mp 106−107 °C). ^{1}H NMR (400 MHz, $CDCl_3$): δ 6.67-6.79 (m, 2H; Ar H), 6.53 (s, 1H; Ar H), 4.25 (q, *J* = 7.2 Hz, 4H; $(CH_2CH_3)_2$), 3.77 (s, 3H; OCH_3), 3.53 (s, 2H; Ar-CH_2), 2.13 (s, 3H; Ar-CH_3), 2.01 (s, 3H; (C=O)CH_3), 1.28 (t, *J* = 7.2 Hz, 6H; $(CH_2CH_3)_2$). ^{13}C NMR (100 MHz, $CDCl_3$): δ 169.12 (NH*C*=O), 167.43 (2C, (*C*=O)OEt), 156.84 (Ar *C*), 131.98 (Ar *C*), 127.99 (Ar *C*), 126.81 (Ar *C*), 126.37 (Ar *C*), 109.52 (Ar *C*), 67.61 (quaternary C), 62.25 (2C, CH_2CH_3), 51.99 (OCH_3), 36.89 (Ar−CH_2), 22.91 ((C=O)CH_3), 16.17 (Ar−CH_3), 14.12 (2C, CH_2CH_3). MS (ESI): *m*/*z* (%): 374.6 ($[M + Na]^+$) (100), 352.6 ($[M + H]^+$) (80). Anal. calcd for $C_{18}H_{25}NO_6$: C 61.52, H 7.17, N 3.99; found: C, 61.52; H, 7.17; N, 3.98.

***m*-Methyl-DL-tyrosine**

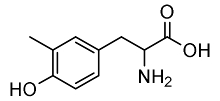

m-Methyl-DL-tyrosine was prepared from diethyl 2-acetamido-2-(4-methoxy-3-methylbenzyl)malonate by the method used for (*R,S*)-3,5-dihydroxyphenylalanine from (*R,S*)-2-amino-3-(3,5-dimethoxyphenyl)propanoic acid, as described in previous reports.[7,8] A solution of diethyl 2-acetamido-2-(4-methoxy-3-methylbenzyl)malonate (1.0 g, 0.0028 mol) in 62% aqueous hydrobromic acid (5.5 mL) and acetic acid (3.0 mL) was heated in a sealed tube at 150 °C for three hours. The mixture was diluted with water (10.0 mL), decolorized with charcoal and concentrated, affording a residue which was recrystallized from water and ethanol to give *m*-methyl-DL-tyrosine as a white solid. Yield 0.24 g, 43%. mp

282−285.4 °C (lit.[18] mp 285 °C decomp.). ^{1}H NMR (400 MHz, D_2O): δ 6.98 (d, *J* = 8.0 Hz, 1H; Ar H), 6.64 (d, *J* = 2.4 Hz, 1H; Ar H), 6.58 (dd, *J* = 8.0, 2.4 Hz, 1H; Ar H), 3.54 (dd, *J* = 8.8, 6.0 Hz, 1H; C*H*), 3.03 (dd, *J* = 14.0, 6.0 Hz, 1H; C*H*HCH), 2.71 (dd, *J* = 14.0, 8.8 Hz, 1H; C*H*HCH), 2.21 (s, 3H; C*H*$_3$). ^{13}C NMR (100 MHz, D_2O): δ 179.02 (*C*=O), 156.71 (Ar *C*), 138.62 (Ar *C*), 131.39 (Ar *C*), 125.61 (Ar *C*), 117.88 (Ar *C*), 113.54 (Ar *C*), 56.05 (*C*H), 35.66 (*C*H$_2$), 18.56 ppm (Ar-*C*H$_3$). MS (ESI): *m/z* (%): 218.4 ([M + Na]$^+$) (100), 196.6 ([M + H]$^+$) (75). Anal. calcd for $C_{10}H_{13}NO_3$: C 61.53, H 6.71, N 7.18; found: C 61.58, H 6.71, N 7.18. The spectral properties of this compound were identical with those previously reported for *m*-methyl-DL-tyrosine.[14]

1-(Bromomethyl)-4-methoxy-2-methylbenzene

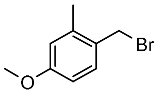

1-(Bromomethyl)-4-methoxy-2-methylbenzene was prepared from 4-methoxy-1,2-dimethylbenzene by the method of Sabol.[19] α,α,α-Trifluorotoluene was used as a substitute for carbon tetrachloride as the solvent for benzylic bromination.[20] To a solution of 4-methoxy-1,2-dimethylbenzene (2.0 g, 0.015 mol) and N-bromosuccinimide (2.86 g, 0.016 mol) in dry α,α,α-trifluorotoluene (50.0 mL), azobisisobutyronitrile (0.07 g) was added. The resultant mixture was heated under reflux for 15 minutes, then cooled, washed with water (3 × 15 mL), dried (sodium sulphate) and concentrated to give a light brown oil. ^{1}H NMR of this crude bromination product mixture showed 1-(bromomethyl)-4-methoxy-2-methylbenzene as the major component. The crude product mixture was used for the following reaction without purification, as described in an earlier report.[19]

Diethyl 2-acetamido-2-(4-methoxy-2-methylbenzyl)malonate

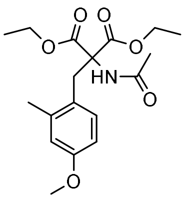

Diethyl 2-acetamido-2-(4-methoxy-2-methylbenzyl)malonate was prepared from 1-(bromomethyl)-4-methoxy-2-methylbenzene by the method of Sabol.[19] To a solution of diethyl acetaminomalonate (3.5 g, 0.016 mol) and sodium hydride (0.39 g, 0.016 mol) in dimethylformamide (20.0 mL), the crude bromination mixture was added. The mixture was stirred at room temperature overnight, then diluted with water (10.0 mL) and extracted with diethyl ether (3 × 10 mL). The combined ether extracts were dried (sodium sulphate) and concentrated, and was recrystallized from dichloromethane and hexane to give diethyl 2-acetamido-2-(4-methoxy-2-methylbenzyl)malonate. The overall yield of the reactions from 4-methoxy-1,2-dimethylbenzene to 1-(bromomethyl)-4-methoxy-2-methylbenzene, and to diethyl 2-acetamido-2-(4-methoxy-2-methylbenzyl)malonate is 2.1 g, 40%. mp 105.3−106.9 °C (lit.[19] mp 102−103 °C). ^{1}H NMR (400 MHz, $CDCl_3$): δ 6.84 (d, *J* = 8.4 Hz, 1H; Ar H), 6.55 (d, *J* = 2.8 Hz, 1H; Ar H), 6.62 (dd, *J* = 8.4, 2.8 Hz, 1H; Ar H), 4.26 (q, *J* = 7.2 Hz, 4H; (C*H*$_2$CH$_3$)$_2$), 3.74 (s, 3H; OC*H*$_3$), 3.61 (s, 2H; Ar-C*H*$_2$), 2.18 (s, 3H; Ar-C*H*$_3$), 2.00 (s, 3H; (C=O)C*H*$_3$), 1.27 (t, *J* = 7.2 Hz, 6H; (CH$_2$C*H*$_3$)$_2$). ^{13}C NMR (100 MHz, $CDCl_3$): δ 169.28 (NH*C*=O), 167.99 (2C, (*C*=O)OEt), 158.46 (Ar *C*), 138.99 (Ar *C*), 131.52 (Ar *C*), 125.51 (Ar *C*), 115.88 (Ar *C*), 111.21 (Ar *C*), 67.10 (quaternary C), 62.64 (2C, *C*H$_2$CH$_3$), 55.15 (O*C*H$_3$), 34.08 (Ar-*C*H$_2$), 23.16 ((C=O)*C*H$_3$), 19.67 (Ar-*C*H$_3$), 14.04 (2C, CH$_2$*C*H$_3$). MS (ESI): *m/z* (%): 374.6 ([M + Na]$^+$) (100), 352.7 ([M + H]$^+$) (60). Anal. calcd for $C_{18}H_{25}NO_6$: C 61.52, H

7.17, N 3.99; found: C, 61.53; H, 7.17; N, 3.98.

o-Methyl-DL-tyrosine

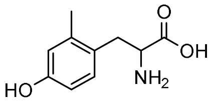

o-Methyl-DL-tyrosine was prepared from diethyl 2-acetamido-2-(4-methoxy-2-methylbenzyl)malonate by the method used for the hydrolysis of (*R,S*)-2-amino-3-(3,5-dimethoxyphenyl)propanoic acid.[7,8] A solution of diethyl 2-acetamido-2-(4-methoxy-2-methylbenzyl)malonate (1.0 g, 0.0032 mol) in 62% aqueous hydrobromic acid (5.5 mL) and acetic acid (3.0 mL) was heated in a sealed tube at 150 °C for three hours. The mixture was diluted with water (10.0 mL), decolorized with charcoal and concentrated, affording a residue which was recrystallized from water and ethanol to give *o*-methyl-DL-tyrosine as white solid. Yield 0.44 g, 71%. mp 246.4−247.1 °C (lit.[19] mp 245−249°C). ^{1}H NMR (400 MHz, D_2O): δ 6.83 (d, J = 8.0 Hz, 1H; Ar H), 6.50 (d, J = 2.4 Hz, 1H; Ar H), 6.45 (dd, J = 8.0, 2.4 Hz, 1H; Ar H), 3.41 (dd, J = 8.8, 6.0 Hz, 1H; C*H*), 2.89 (dd, J = 14.0, 6.0 Hz, 1H; C*H*HCH), 2.56 (dd, J = 14.0, 8.8 Hz, 1H; C*H*HCH), 2.07 (s, 3H; CH_3). ^{13}C NMR (100 MHz, D_2O): δ 178.63 (*C*=O), 156.47 (Ar *C*), 138.31 (Ar *C*), 131.45 (Ar *C*), 125.32 (Ar *C*), 118.01 (Ar *C*), 113.24 (Ar *C*), 55.89 (*C*H), 35.36 (*C*H_2), 17.26 (*C*H_3). MS (ESI): *m/z* (%): 218.4 ([M + Na]$^+$) (100), 196.8 ([M + H]$^+$) (40). Anal. calcd for $C_{10}H_{13}NO_3$: C 61.53, H 6.71, N 7.18; found: C 61.53, H 6.70, N 7.18. The spectral properties of this compound were identical with those previously reported for *o*-methyl-DL-tyrosine.[14]

1-(Bromomethyl)-4-methoxy-2-nitrobenzene

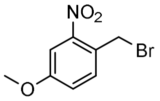

1-(Bromomethyl)-4-methoxy-2-nitrobenzene was prepared from 4-methoxy-1-methyl-2-nitrobenzene by the method of Katritzky.[21] Again α,α,α-trifluorotoluene was used as solvent for the bromination of 4-methoxy-1-methyl-2-nitrobenzene.[20] To a solution of 4-methoxy-1-methyl-2-nitrobenzene (2.0 g, 0.012 mol) and N-bromosuccinimide (2.33 g, 0.013 mol) in dry α,α,α-trifluorotoluene (40.0 mL), azobisisobutyronitrile (0.06 g) was added. The resultant mixture was refluxed under strong illumination using a 400-W General Electric sunlamp for six hours, then cooled and filtered. The filtrate was concentrated to yield a light brown oil which was purified with chromatography from benzene and hexane to give 1-(bromomethyl)-4-methoxy-2-nitrobenzene as light yellow crystals. Yield 1.33g, 45%. mp 64.1−65.2 °C (lit.[21] mp 63−64 °C). ^{1}H NMR (400 MHz, $CDCl_3$): δ 7.52-7.53 (m, 1H; Ar H), 7.42-7.45 (m, 1H; Ar H), 7.09-7.13 (m, 1H; Ar H), 4.78 (s, 2H; CH_2), 3.87 (s, 3H; CH_3). ^{13}C NMR (101 MHz, $CDCl_3$): δ 160.15 (Ar *C*), 148.62 (Ar *C*), 133.68 (Ar *C*), 124.75 (Ar *C*), 119.91 (Ar C), 110.52 (Ar C), 56.08 (*C*H_3), 29.30 ppm (*C*H_2). MS (ESI): *m/z* (%): 269.6 (C_9H_{11}) (100), 248.2 ([M + H]$^+$) (30). The spectral properties of this compound were identical with those previously reported for 1-(bromomethyl)-4-methoxy-2-nitrobenzene.[21]

Diethyl 2-acetamido-2-(4-methoxy-2-nitrobenzyl)malonate

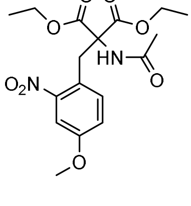

Diethyl 2-acetamido-2-(4-methoxy-2-nitrobenzyl)malonate was prepared from 1-(bromomethyl)-4-methoxy-2-nitrobenzene by the method of McCord.[13] To a solution of metallic sodium (0.1 g, 0.0045 mol) in dry ethanol (10.0 mL), diethyl acetaminomalonate (0.97 g, 0.0045 mol) was added. The resultant mixture was stirred at room temperature for 30 minutes, mixed with 1-(bromomethyl)-4-methoxy-2-nitrobenzene (1.0 g, 0.004 mol), heated under reflux for three hours, then filtered. The filtrate was cooled and kept at 0 °C overnight. The precipitate was recrystallized from ethanol to give diethyl 2-acetamido-2-(4-methoxy-2-nitrobenzyl)malonate as white solid. Yield 1.02 g, 66%. mp 110.1−111.7 °C (lit.[13] mp 108−109 °C). ^{1}H NMR (400 MHz, $CDCl_3$): δ 7.32 (d, *J* = 2.4 Hz, 1H; Ar H), 7.13 (d, *J* = 8.4 Hz, 1H; Ar H), 7.01 (dd, *J* = 8.4, 2.4 Hz, 1H; Ar H), 6.45 (broad absorption, 1H; N*H*), 4.23 (q, *J* = 7.2 Hz, 4H; $(CH_2CH_3)_2$), 3.97 (s, 2H; Ar-CH_2), 3.83 (s, 3H; OCH_3), 1.95 (s, 3H; (C=O)CH_3), 1.26 (t, *J* = 7.2 Hz, 6H; $(CH_2CH_3)_2$). ^{13}C NMR (100 MHz, $CDCl_3$): δ 169.49 (NH*C*=O), 167.64 (2C, (*C*=O)OEt), 159.23 (Ar *C*), 150.99 (Ar *C*), 134.72 (Ar *C*), 121.61 (Ar *C*), 119.01 (Ar *C*), 109.74 (Ar *C*), 66.48 (quaternary C), 62.99 (2C, CH_2CH_3), 55.90 (OCH_3), 33.83 (Ar-CH_2), 22.94 ((C=O)CH_3), 13.99 (2C; CH_2CH_3).MS (ESI): *m*/*z* (%): 405.3 ([M + Na]$^+$) (100), 383.3 ([M + H]$^+$) (10). Anal. calcd for $C_{17}H_{22}N_2O_8$: C 53.40, H 5.80, N 7.33; found: C 53.41, H 5.78, N 7.37.

***o*-Nitro-DL-tyrosine**

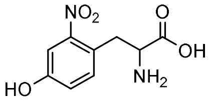

o-Nitro-DL-tyrosine was prepared from diethyl 2-acetamido-2-(4-methoxy-2-nitrobenzyl)malonate by the method of McCord.[13] A solution of diethyl 2-acetamido-2-(4-methoxy-2-nitrobenzyl)malonate (1.0 g, 0.0026 mol) in 62% aqueous hydrobromic acid (5.5 mL) and acetic acid (3.0 mL) was heated in a sealed tube at 150 °C for three hours. The mixture was diluted with water (10.0 mL), decolorized with charcoal and concentrated, affording a residue which was recrystallized from water and ethanol to give *o*-nitro-DL-tyrosine as white solid. Yield 0.46 g, 77%. mp 213.3−231.8 °C decomp. (lit.[13] mp 225-231 °C decomp.). ^{1}H NMR (400 MHz, CD_3OD): δ 7.31 (d, *J* = 2.8 Hz, 1H; Ar H), 7.18 (d, *J* = 8.4 Hz, 1H; Ar H), 6.97 (dd, *J* = 8.4, 2.8 Hz, 1H; Ar H), 4.11 (m, 1H; C*H*), 3.35 (m, 1H; C*H*HCH), 3.16 (m, 1H; C*H*HCH). ^{13}C NMR (100 MHz, CD_3OD): δ 170.37 (*C*=O), 157.27 (Ar *C*), 149.40 (Ar *C*), 134.32 (Ar *C*), 121.31 (Ar *C*), 120.40 (Ar *C*), 111.83 (Ar *C*), 53.25 (*C*H), 33.10 (*C*H_2). MS (ESI): *m*/*z* (%): 227.4 ([M + H]$^+$) (90), 249.4 ([M + Na]$^+$) (20), 181.3 (100). Anal. calcd for $C_9H_{10}N_2O_5$: C 47.79, H 4.46, N 12.38; found: C 47.66, H 4.53, N 12.30.

3. Compound characterization

Diethyl 2-acetamido-2-(3-methoxy-5-methylbenzyl)malonate

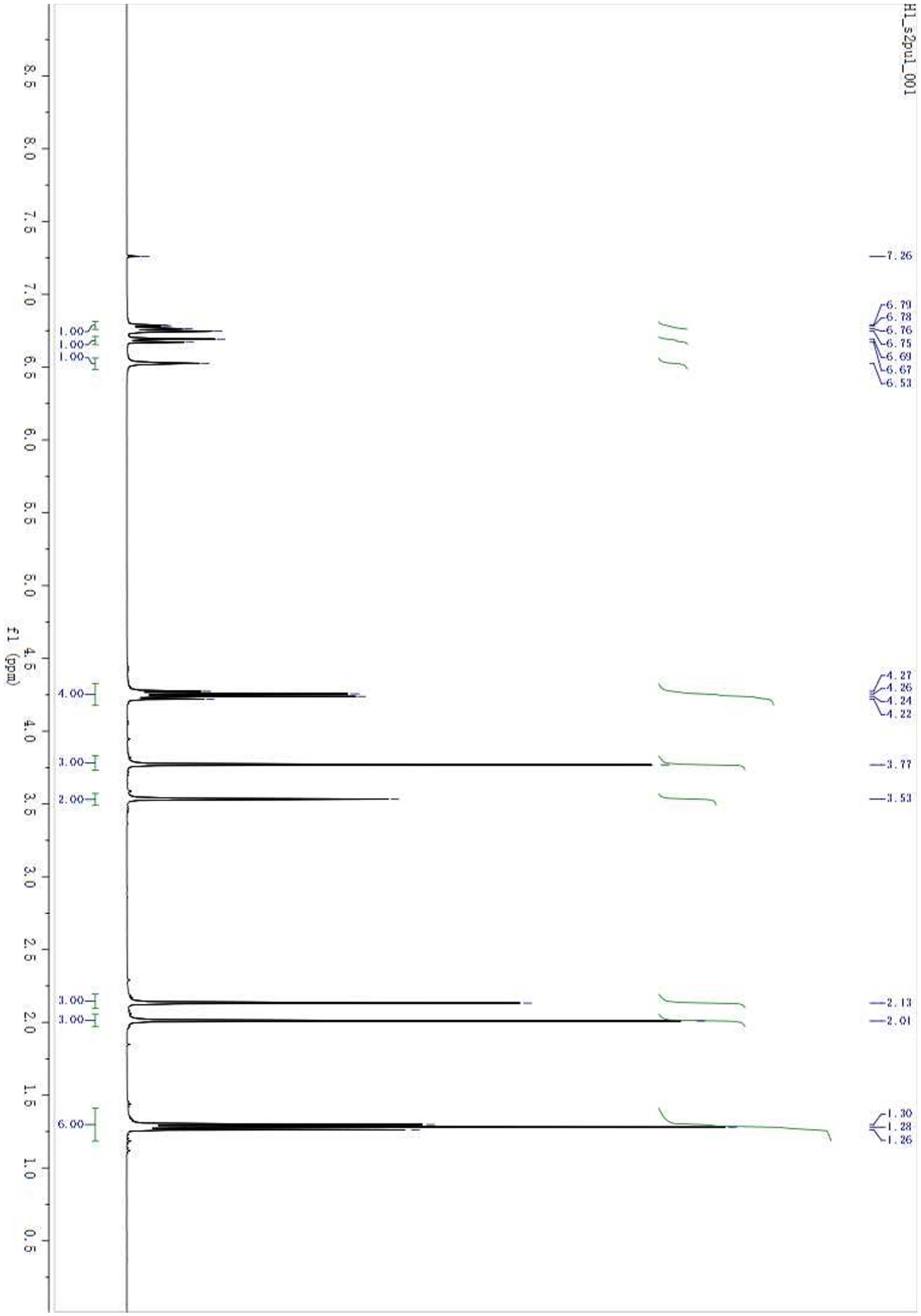
H1_s2pu1_001
7.26
6.79
6.78
6.76
6.75
6.69
6.67
6.53
4.27
4.26
4.24
4.22
3.77
3.53
2.13
2.01
1.30
1.28
1.26
1.00
1.00
1.00
4.00
3.00
2.00
3.00
3.00
6.00
f1 (ppm)

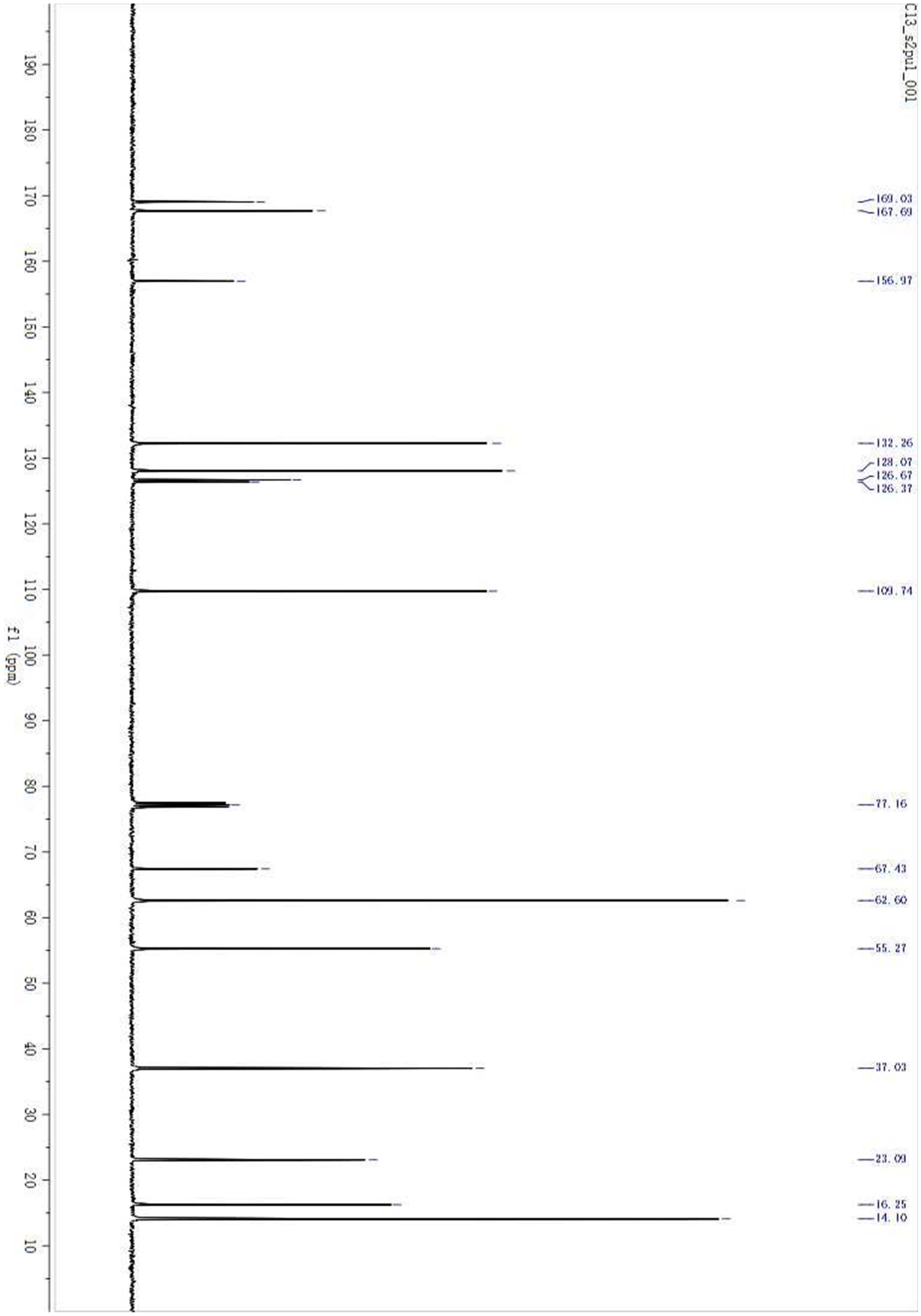
C13_s2pul_001
169.03
167.69
156.97
132.26
128.07
126.67
126.37
109.74
77.16
67.43
62.60
55.27
37.03
23.09
16.25
14.10
f1 (ppm)

Elemental Composition Report

Single Mass Analysis

Tolerance = 3.0 PPM / DBE: min = -1.5, max = 20.0
Element prediction: Off
Number of isotope peaks used for i-FIT = 3

Monoisotopic Mass, Even Electron Ions
25 formula(e) evaluated with 1 results within limits (all results (up to 1000) for each mass)
Elements Used:
C: 0-18 H: 0-27 N: 0-1 O: 0-6 23Na: 0-1

ZQW 2-91malonate/AJ KE375 06-Nov-2012 11:39:43
21651
1457 18 (0.803) Cm (16:18) 1: TOF MS ES+ 1.97e+005

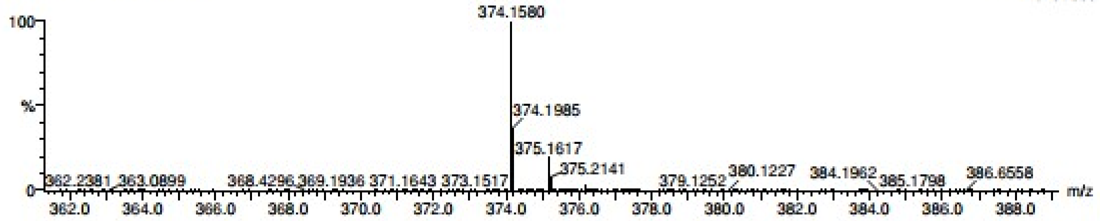

Minimum:				-1.5		
Maximum:		5.0	3.0	20.0		
Mass	Calc. Mass	mDa	PPM	DBE	i-FIT	Formula
374.1580	374.1580	0.0	0.0	6.5	106.1	C18 H25 N O6 23Na

Elemental Composition Report

Single Mass Analysis

Tolerance = 3.0 PPM / DBE: min = -1.5, max = 20.0
Element prediction: Off
Number of isotope peaks used for i-FIT = 3

Monoisotopic Mass, Even Electron Ions
12 formula(e) evaluated with 1 results within limits (all results (up to 1000) for each mass)
Elements Used:
C: 0-18 H: 0-27 N: 0-1 O: 0-6

ZQW 2-91malonate/AJ KE375 06-Nov-2012 11:39:43
21651
1457 20 (0.871) Cm (20:23) 1: TOF MS ES+ 2.52e+004

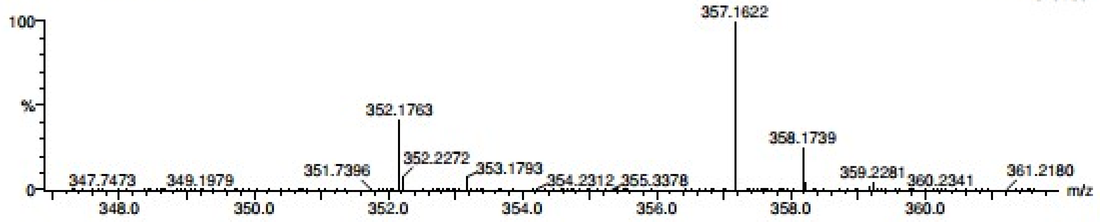

Minimum:				-1.5		
Maximum:		5.0	3.0	20.0		
Mass	Calc. Mass	mDa	PPM	DBE	i-FIT	Formula
352.1763	352.1760	0.3	0.9	6.5	44.8	C18 H26 N O6

3-Hydroxy-5-methyl-DL-phenylalanine

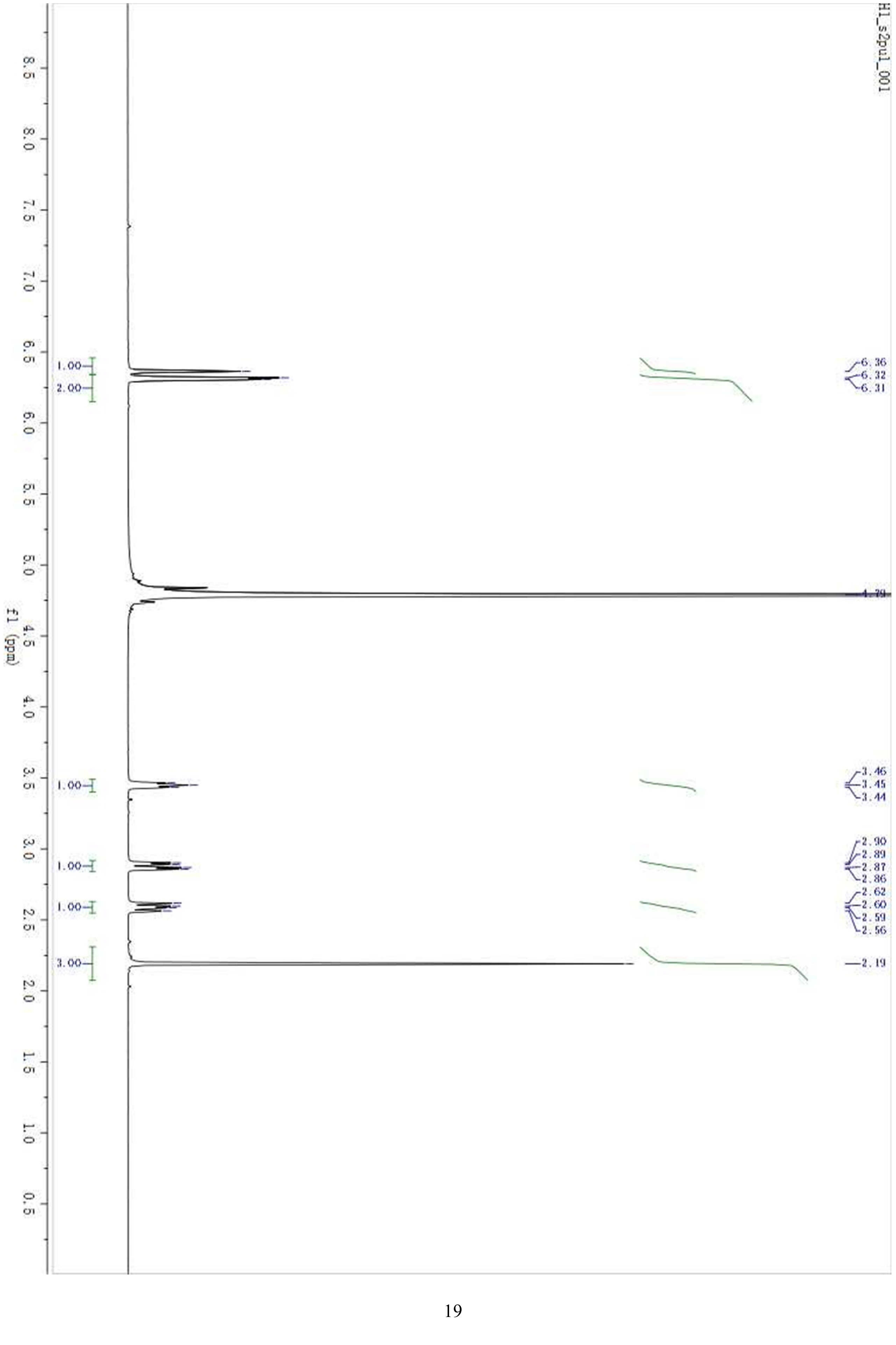

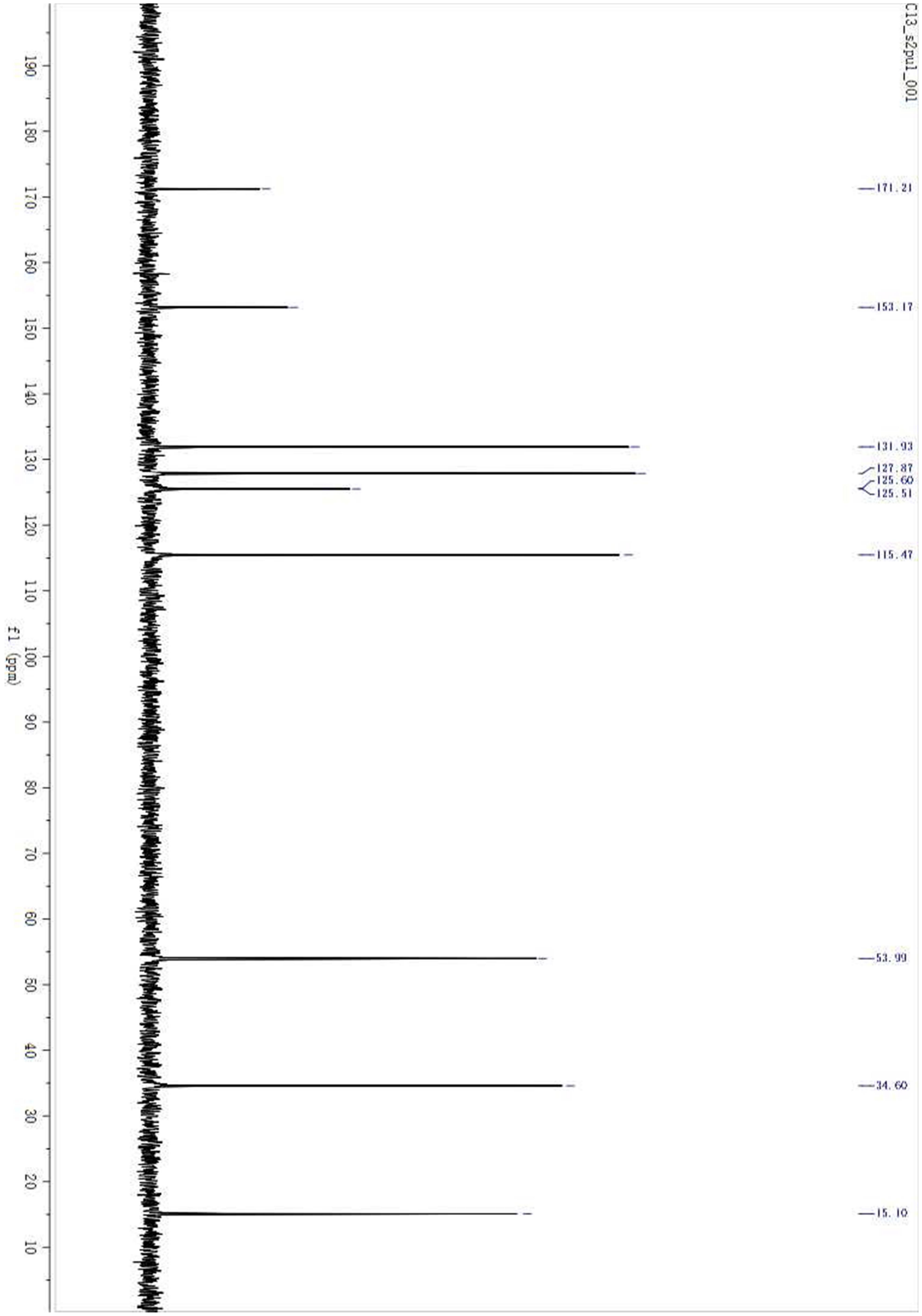
C13_s2pul_001
171.21
153.17
131.93
127.87
125.60
125.51
115.47
53.99
34.60
15.10
f1 (ppm)

Elemental Composition Report **Page 1**

Single Mass Analysis
Tolerance = 3.0 PPM / DBE: min = -1.5, max = 20.0
Element prediction: Off
Number of isotope peaks used for i-FIT = 3

Monoisotopic Mass, Even Electron Ions
75 formula(e) evaluated with 1 results within limits (all results (up to 1000) for each mass)
Elements Used:
C: 0-50 H: 0-60 N: 0-2 O: 0-3 23Na: 0-1
ZQW 2-95/EO KE375 06-Nov-2012 11:58:24
21653
1452 29 (1.294) Cm (26:29) 1: TOF MS ES+ 1.29e+005

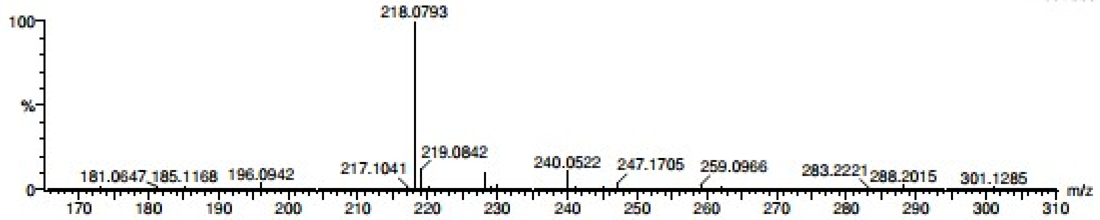

Minimum:				-1.5		
Maximum:		5.0	3.0	20.0		
Mass	Calc. Mass	mDa	PPM	DBE	i-FIT	Formula
218.0793	218.0793	0.0	0.0	4.5	39.8	C10 H13 N O3 23Na

4. ESI Mass spectrometry of purified proteins

Purified protein solutions (50 μL) were analyzed by ESI mass spectrometry by direct injection into the spectrometer running a mobile phase of a 50:50 (v/v) solution of 0.1% formic acid in acetonitrile: 0.1% aqueous formic acid. Deconvoluted mass spectra of PpiB synthesized in the presence of Phe and Tyr analogues are shown below.

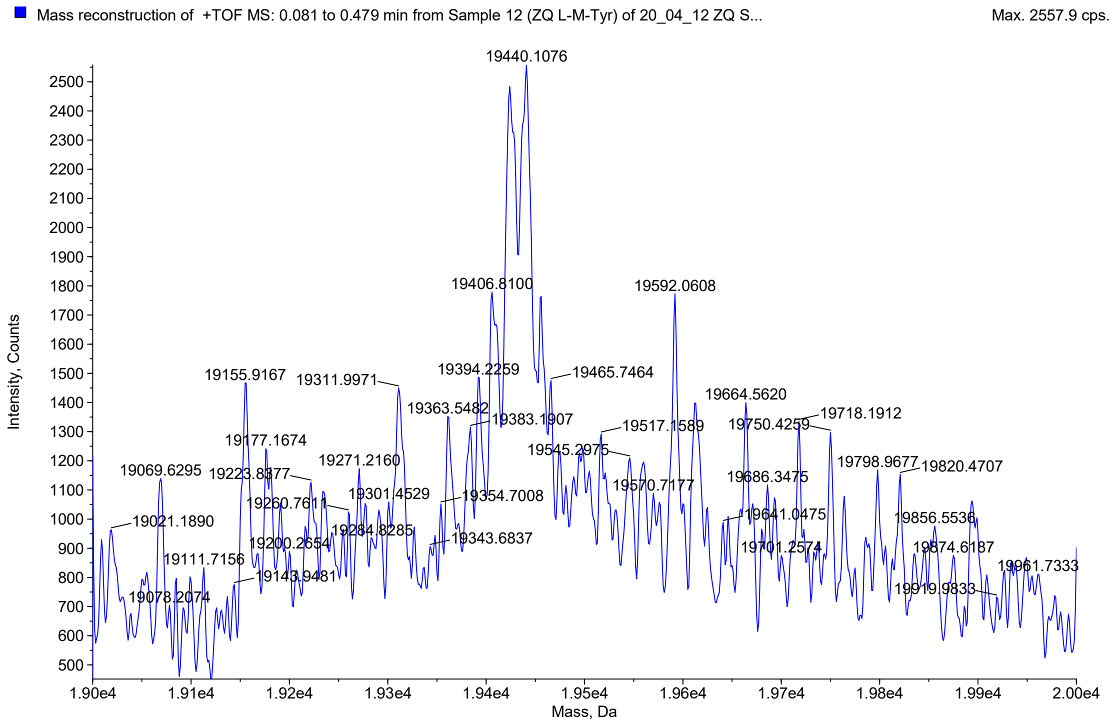

Figure S1. Mass spectral analysis of His$_6$-PpiB synthesized in the presence of *m*-tyrosine (**1b**).

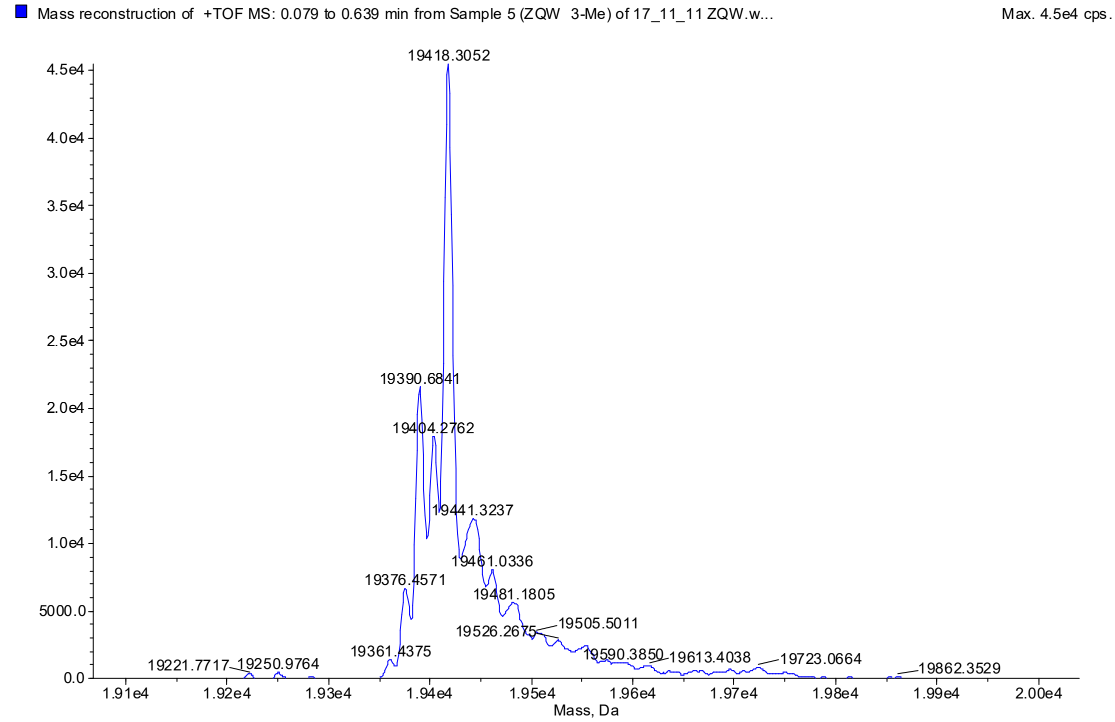

Figure S2. Mass spectral analysis of His$_6$-PpiB synthesized in the presence of *m*-methylphenylalanine (**2b**).

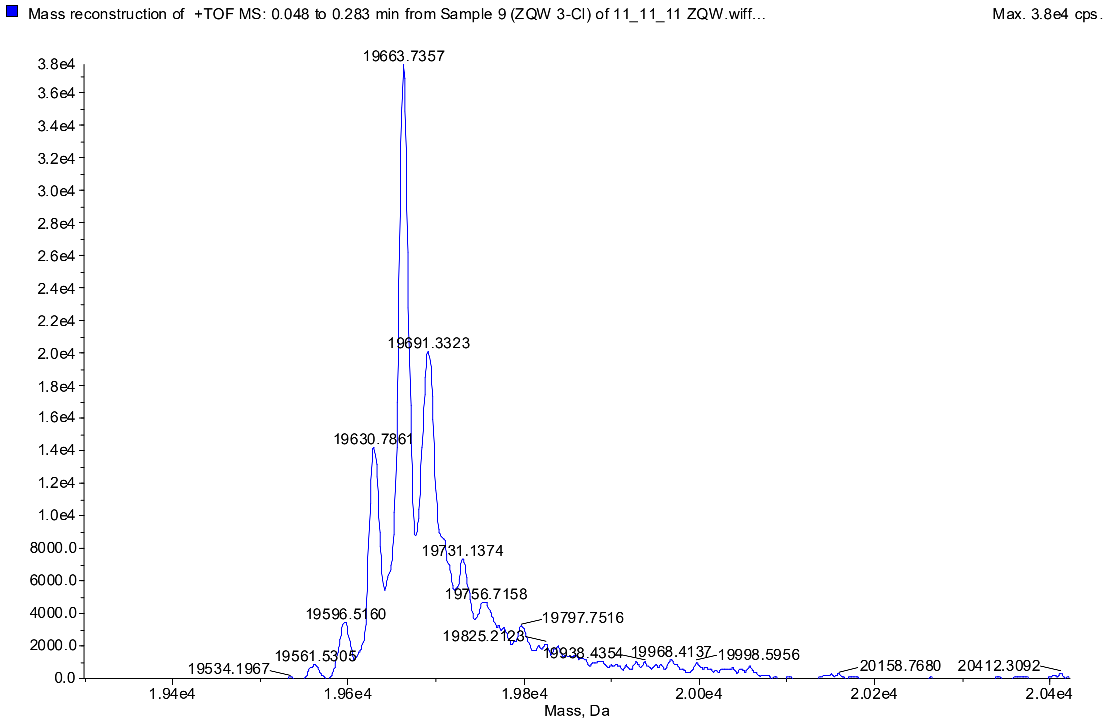

Figure S3. Mass spectral analysis of His$_6$-PpiB synthesized in the presence of *m*-chlorophenylalanine (**3b**).

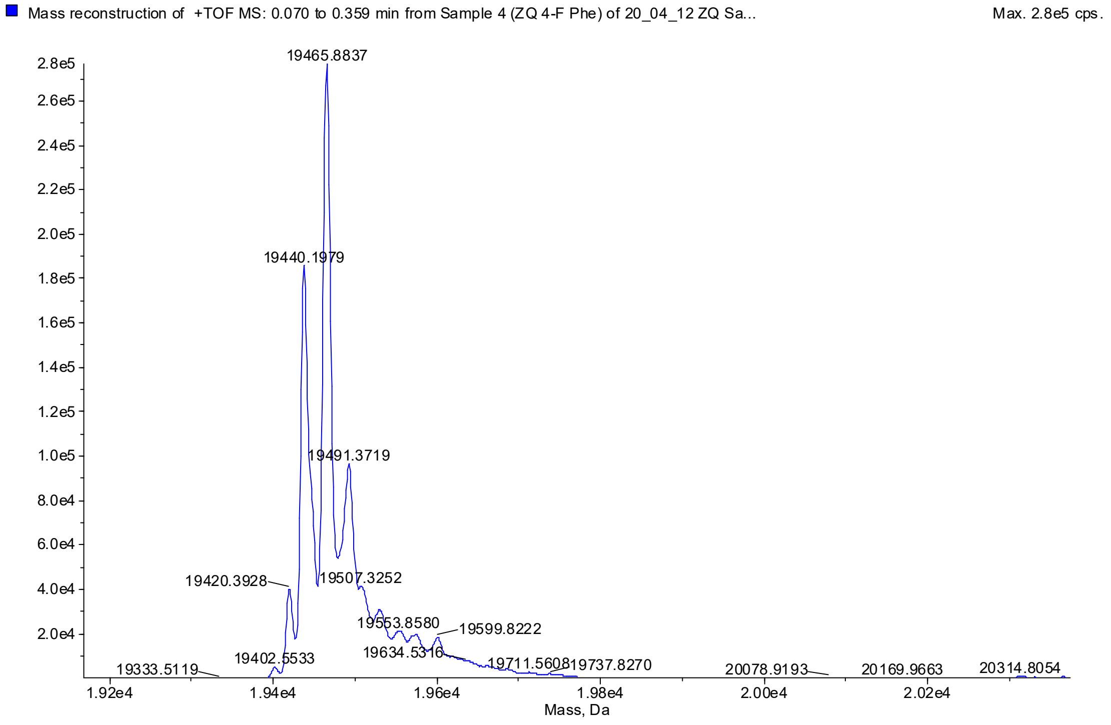

Figure S4. Mass spectral analysis of His$_6$-PpiB synthesized in the presence of *p*-fluorophenylalanine (**5a**).

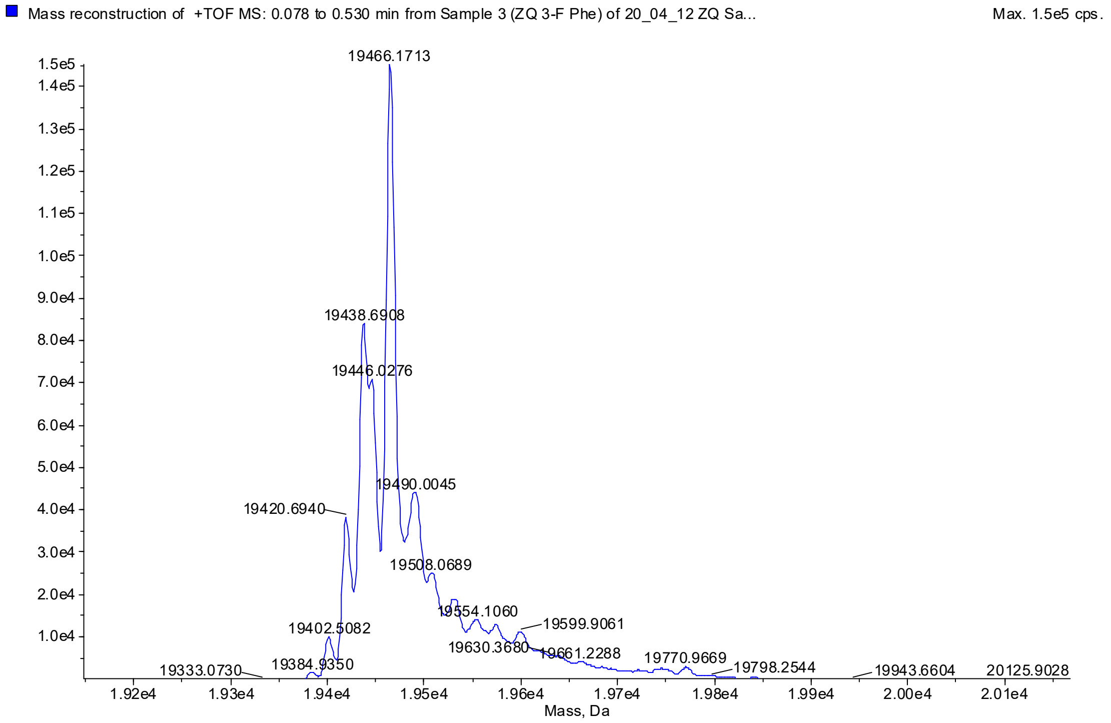

Figure S5. Mass spectral analysis of His$_6$-PpiB synthesized in the presence of *m*-fluorophenylalanine (**5b**).

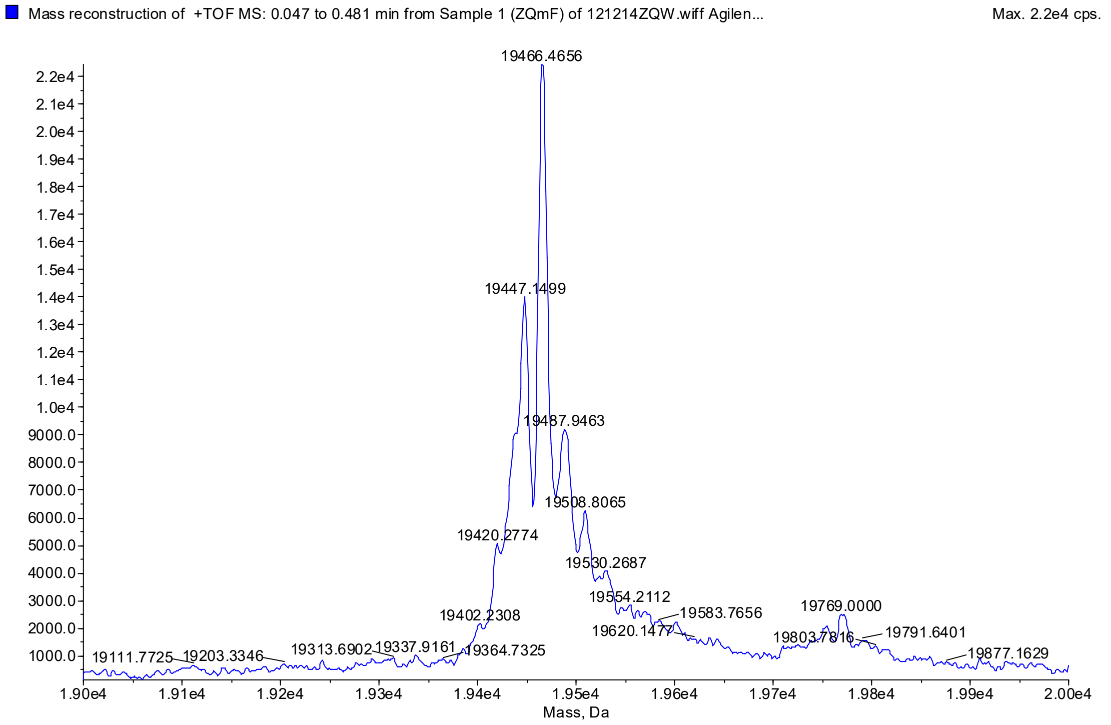

Figure S6. Mass spectral analysis of His$_6$-PpiB synthesized in the presence of *o*-fluorophenylalanine (**5c**).

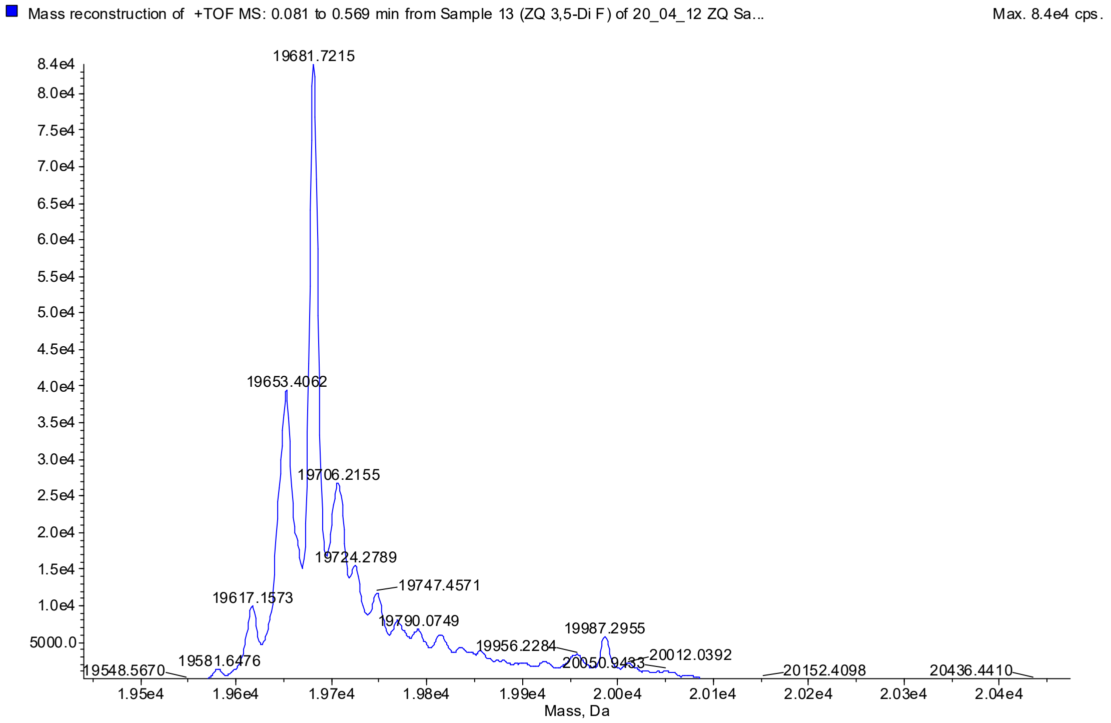

Figure S7. Mass spectral analysis of His$_6$-PpiB synthesized in the presence of 3,5-difluorophenylalanine (**6**).

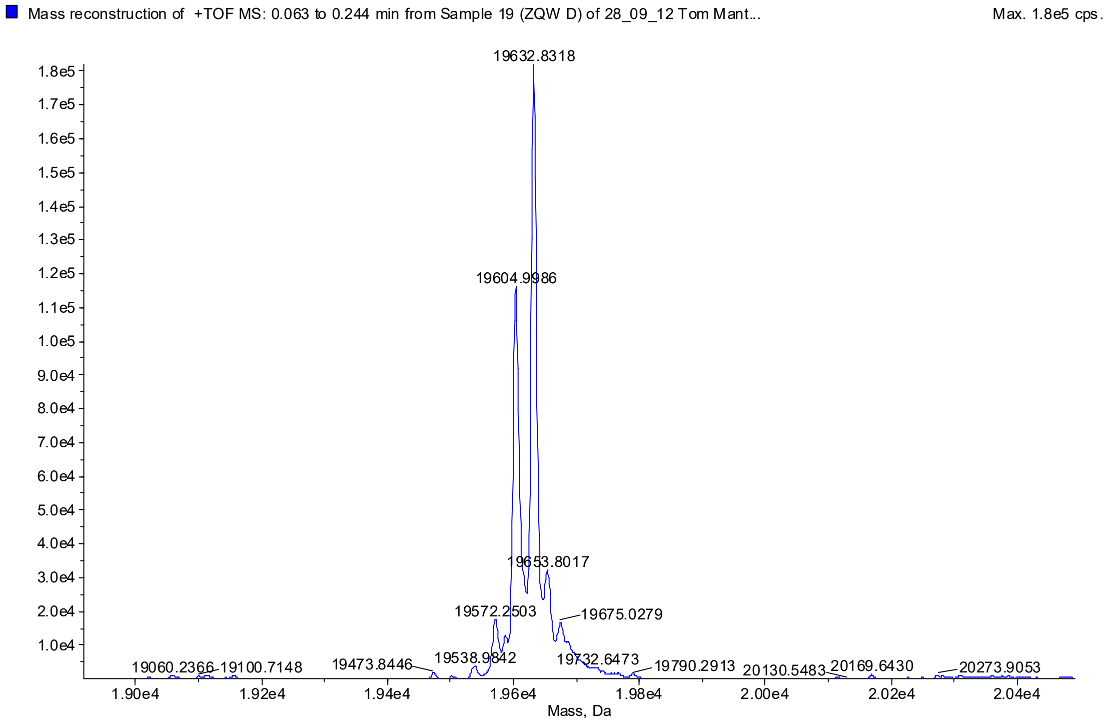

Figure S8. Mass spectral analysis of His$_6$-PpiB synthesized in the presence of 3,5-dihydroxyphenylalanine (**9**).

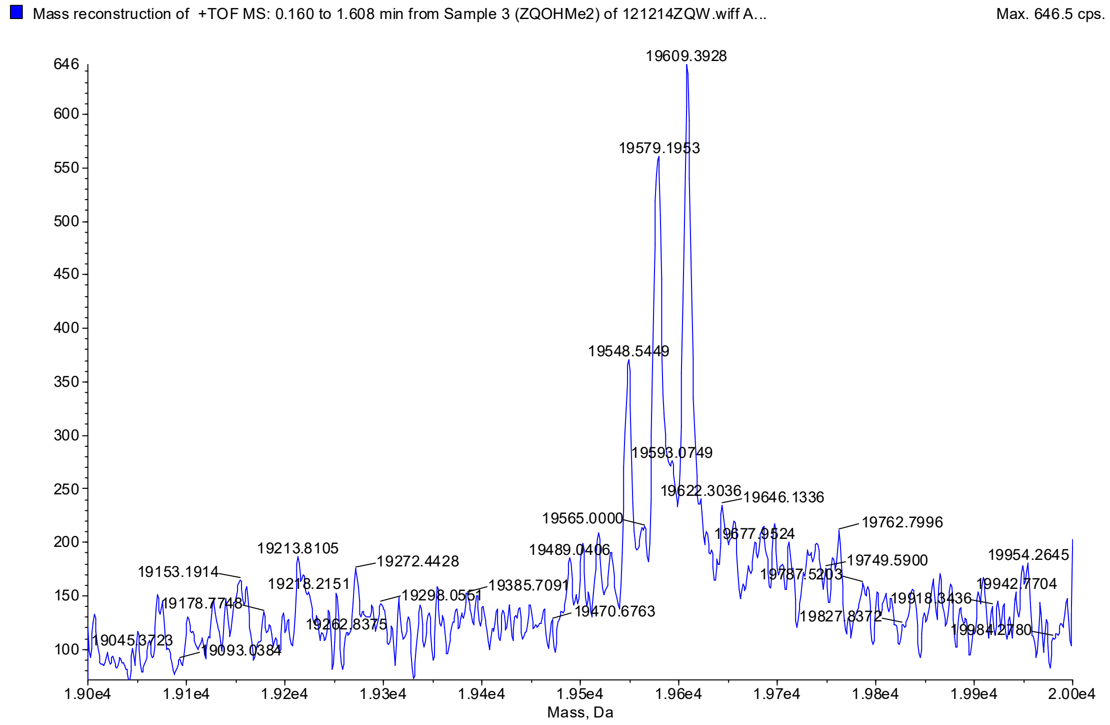

Figure S9. Mass spectral analysis of His$_6$-PpiB synthesized in the presence of 3-hydroxy-5-methylphenylalanine (**10**).

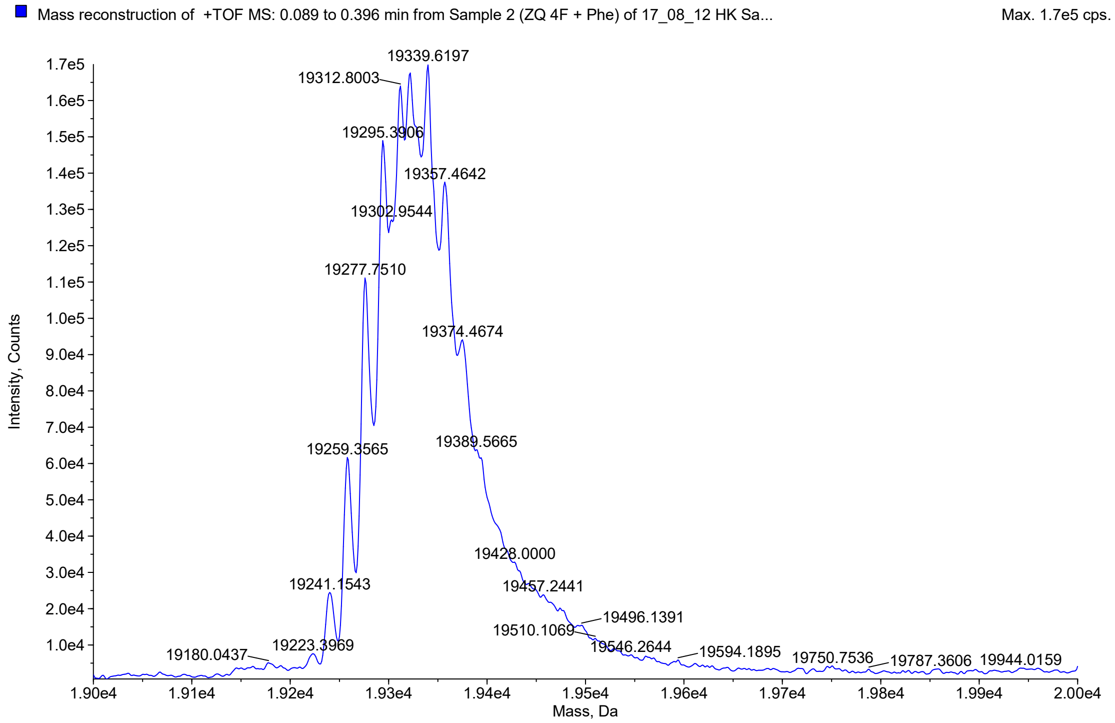

Figure S10. Mass spectral analysis of His$_6$-PpiB synthesized in the presence of 0.2 mM phenylalanine and 2.0 mM *p*-fluorophenylalanine (**5a**).

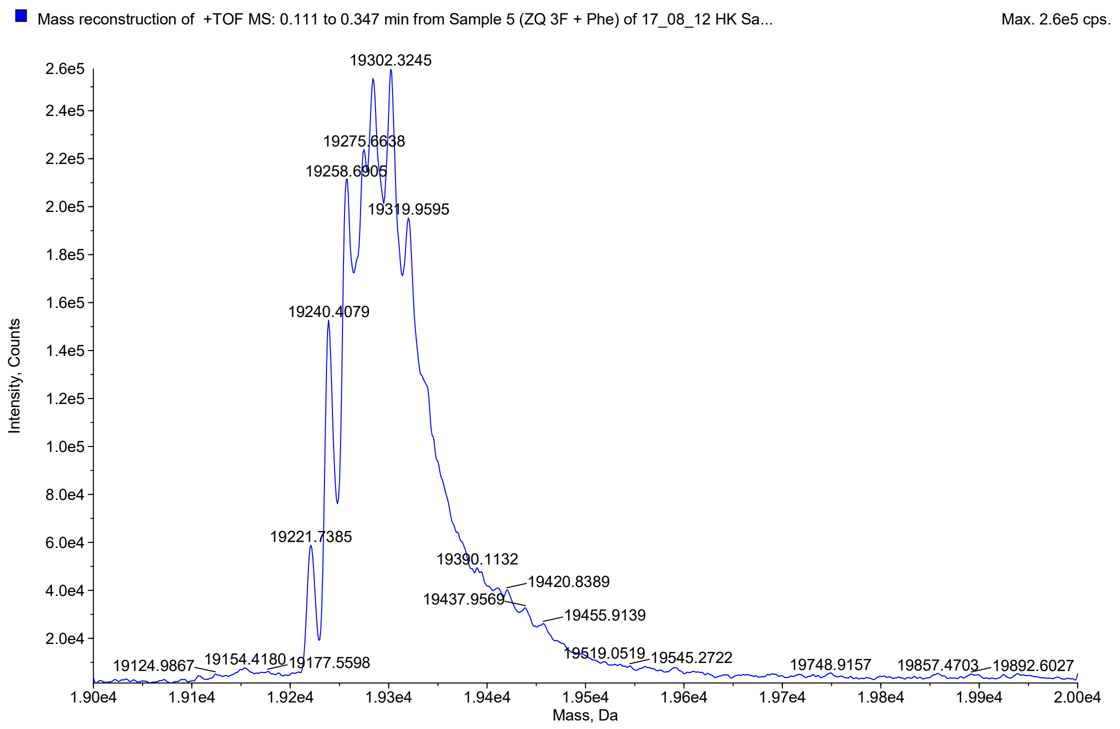

Figure S11. Mass spectral analysis of His$_6$-PpiB synthesized in the presence of 0.2 mM phenylalanine and 2.0 mM *m*-fluorophenylalanine (**5b**).

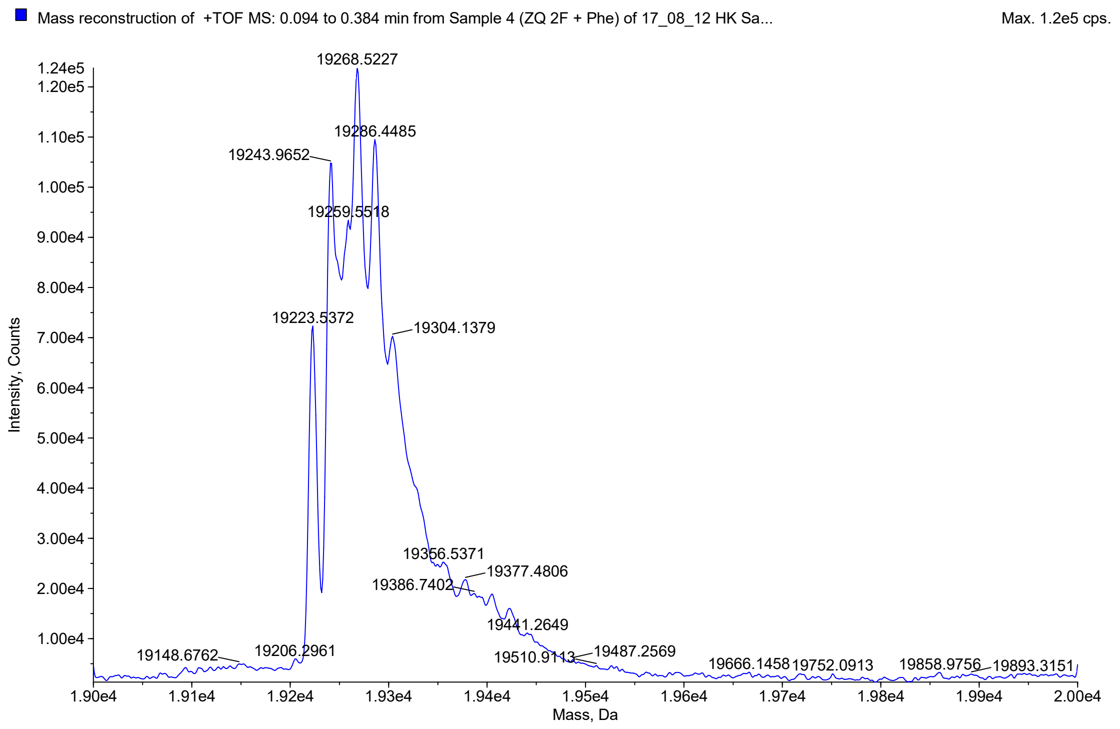

Figure S12. Mass spectral analysis of His$_6$-PpiB synthesized in the presence of 0.2 mM phenylalanine and 2.0 mM *o*-fluorophenylalanine (**5c**).

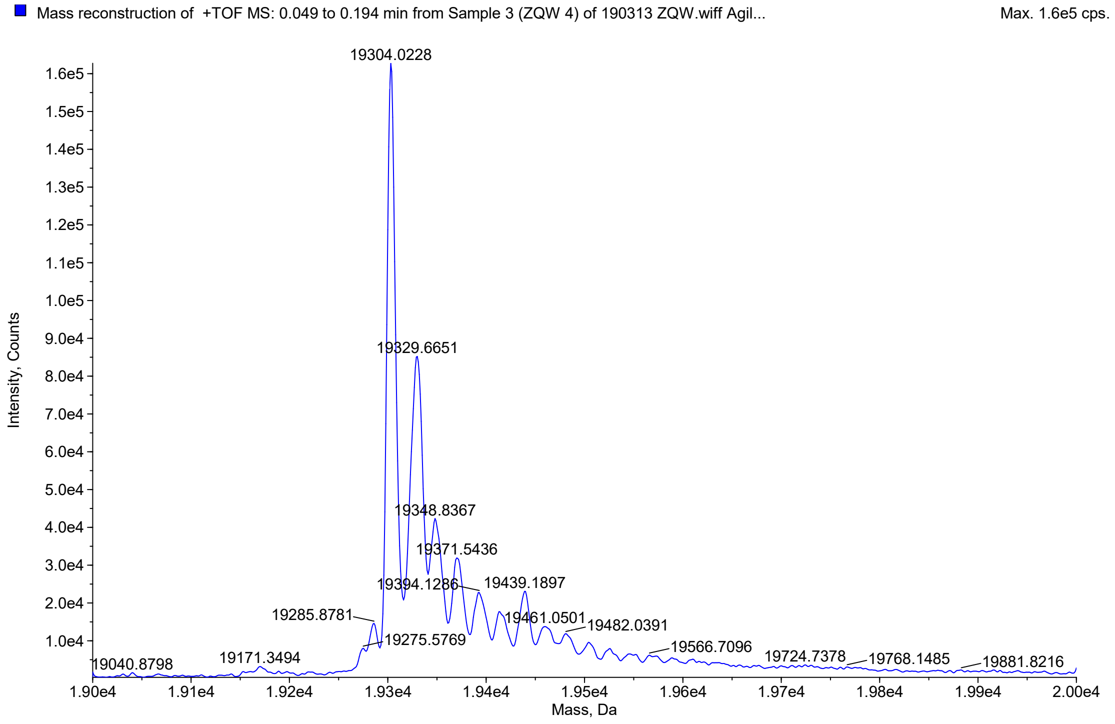

Figure S13. Mass spectral analysis of His$_6$-PpiB synthesized in the presence of *m*-fluorotyrosine (**15a**).

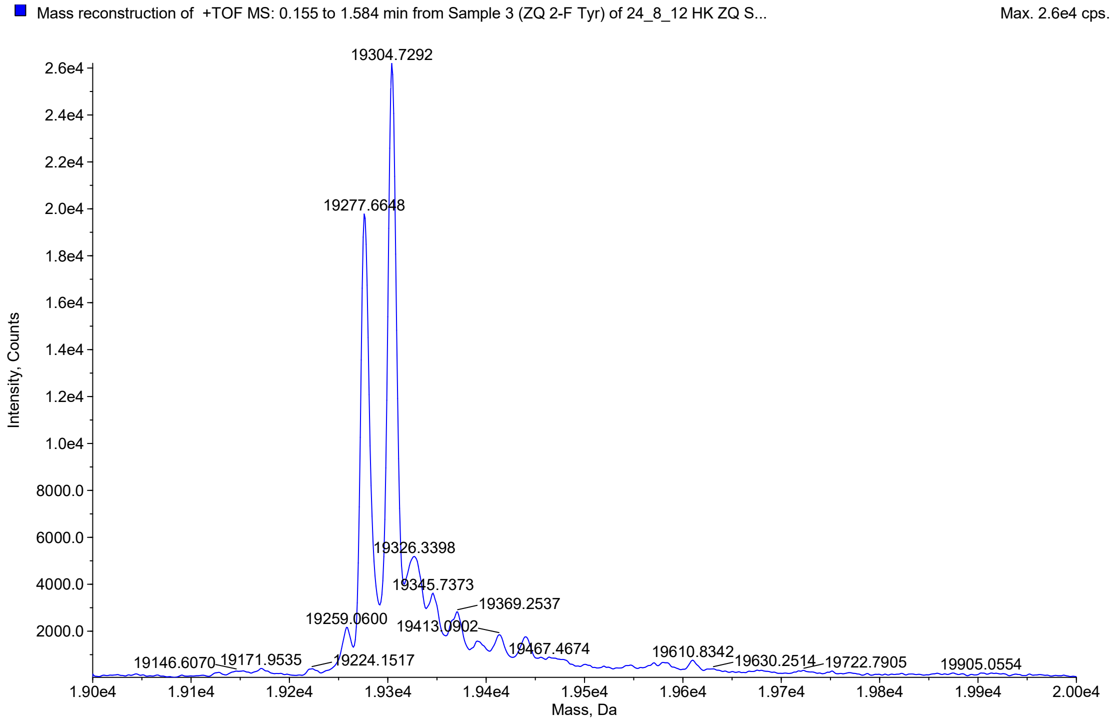

Figure S14. Mass spectral analysis of His$_6$-PpiB synthesized in the presence of *o*-fluorotyrosine (**15b**).

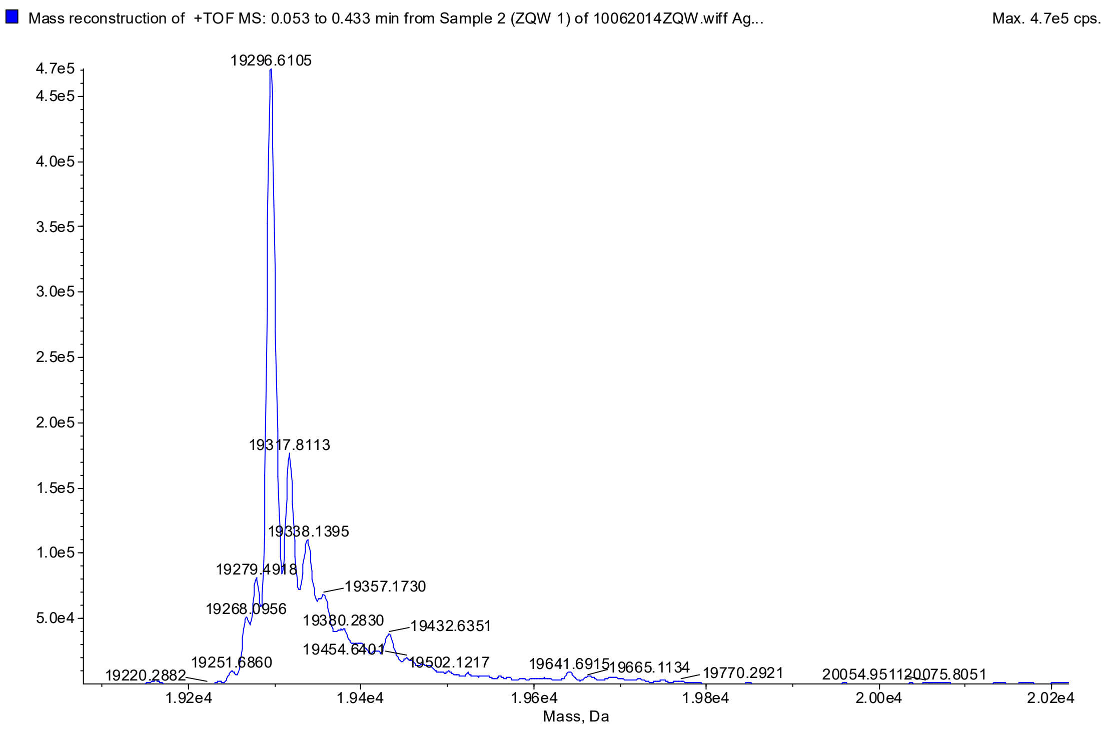

Figure S15. Mass spectral analysis of His$_6$-PpiB synthesized in the presence of 3,4-dihydroxyphenylalanine (**16a**).

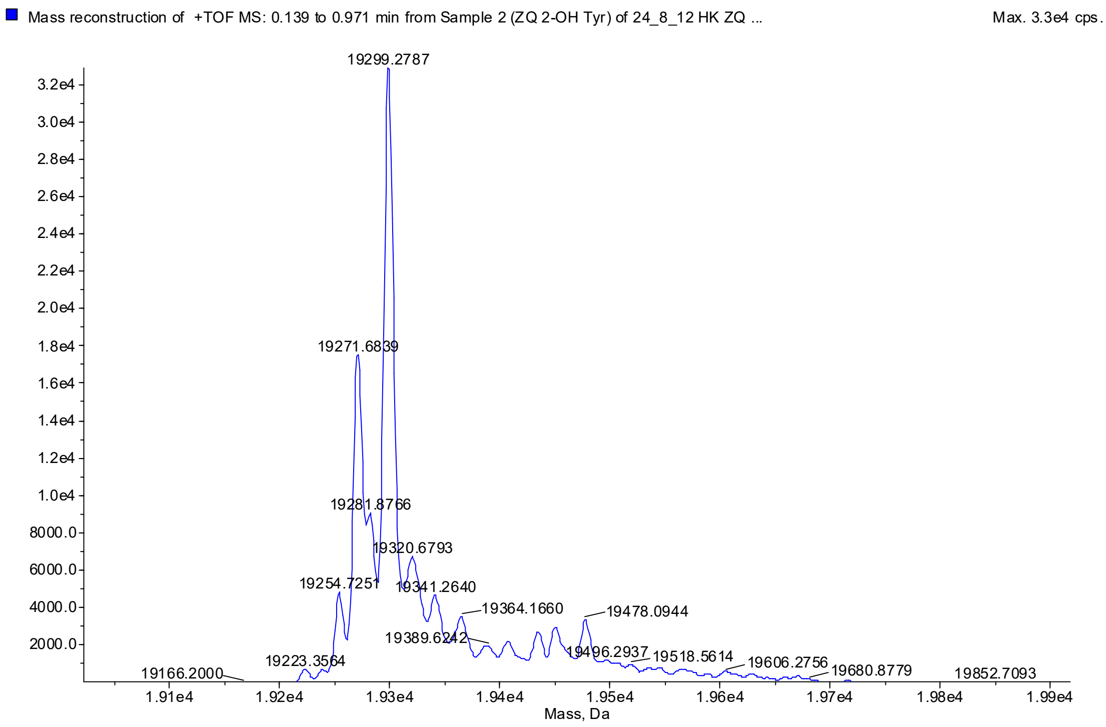

Figure S16. Mass spectral analysis of His$_6$-PpiB synthesized in the presence of 2,4-dihydroxyphenylalanine (**16b**).

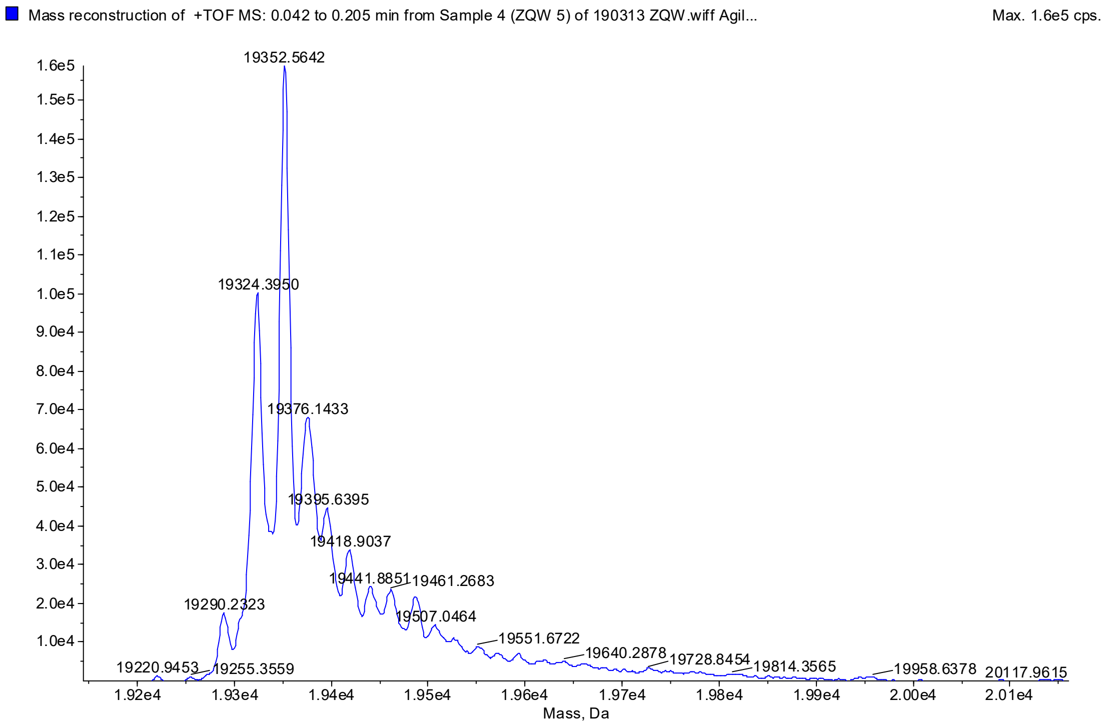

Figure S17. Mass spectral analysis of His$_6$-PpiB synthesized in the presence of *m*-chlorotyrosine (**17a**).

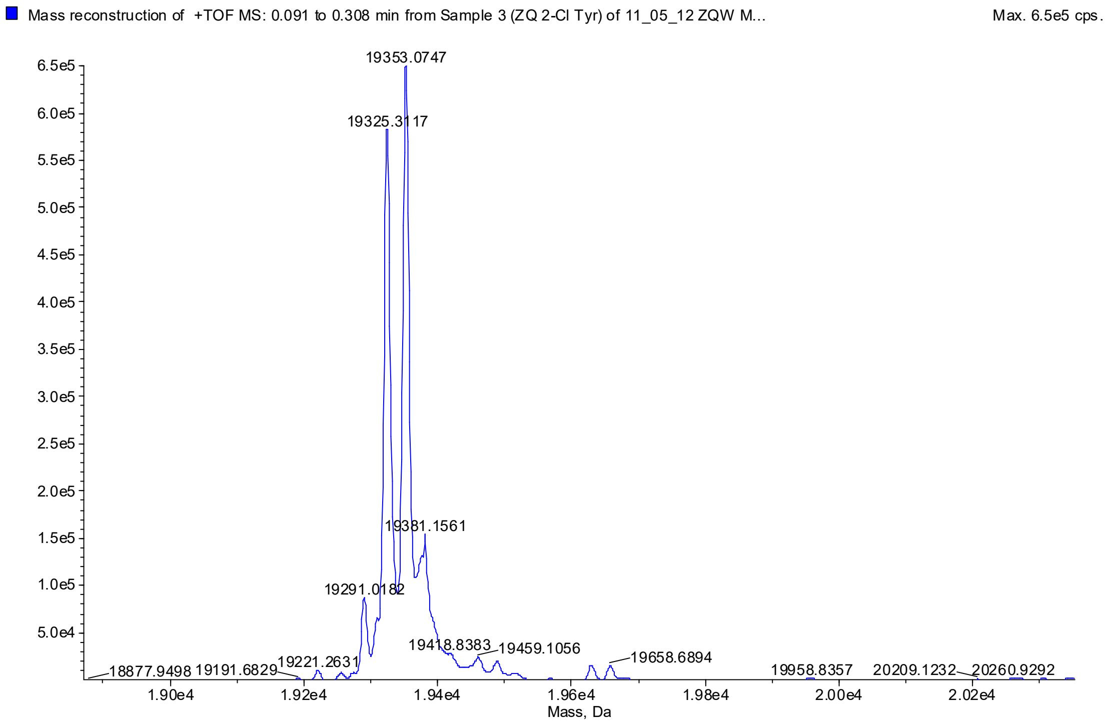

Figure S18. Mass spectral analysis of His$_6$-PpiB synthesized in the presence of *o*-chlorotyrosine (**17b**).

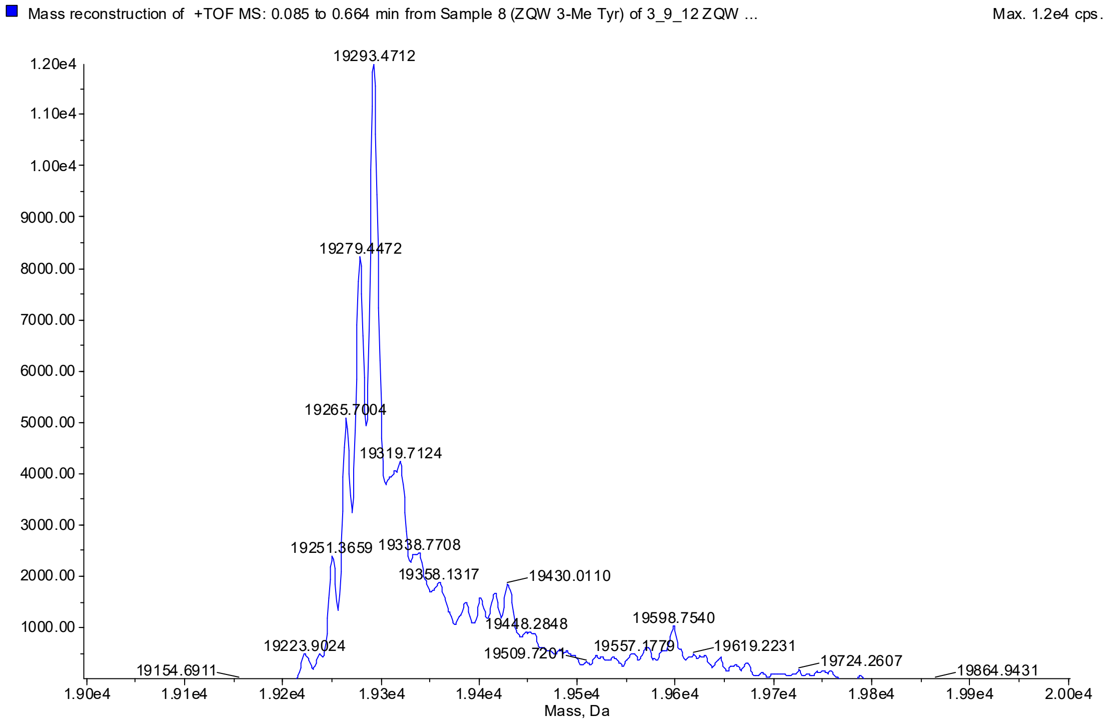

Figure S19. Mass spectral analysis of His$_6$-PpiB synthesized in the presence of *m*-methyltyrosine (**18a**).

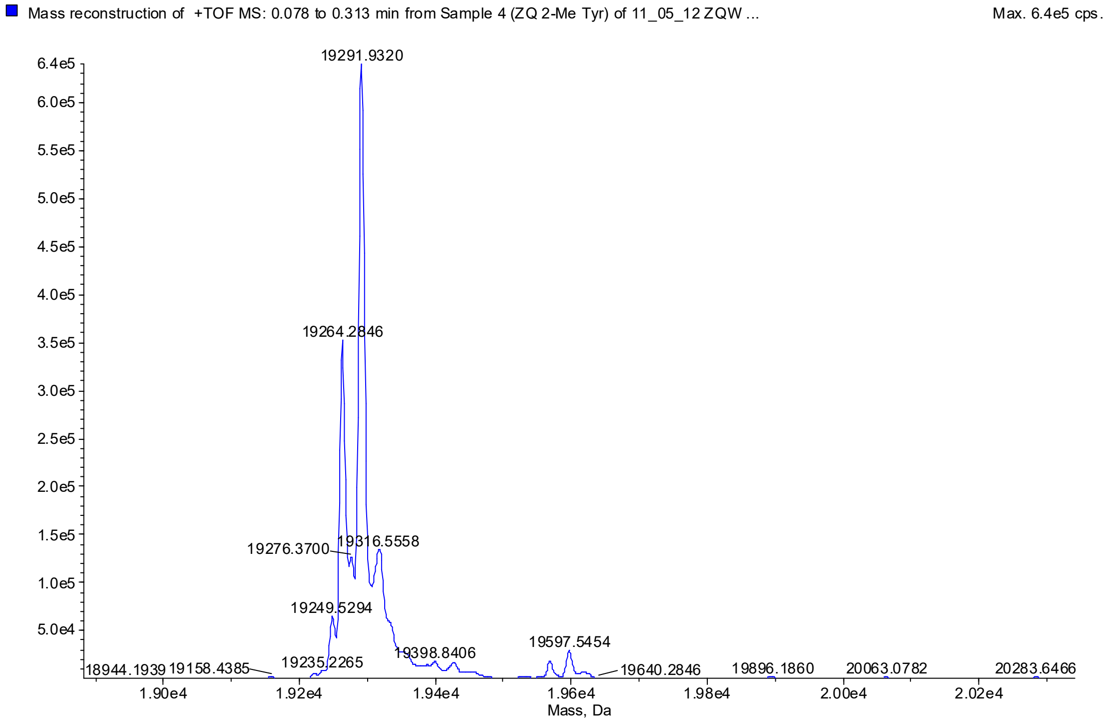

Figure S20. Mass spectral analysis of His$_6$-PpiB synthesized in the presence of *o*-methyltyrosine (**18b**).

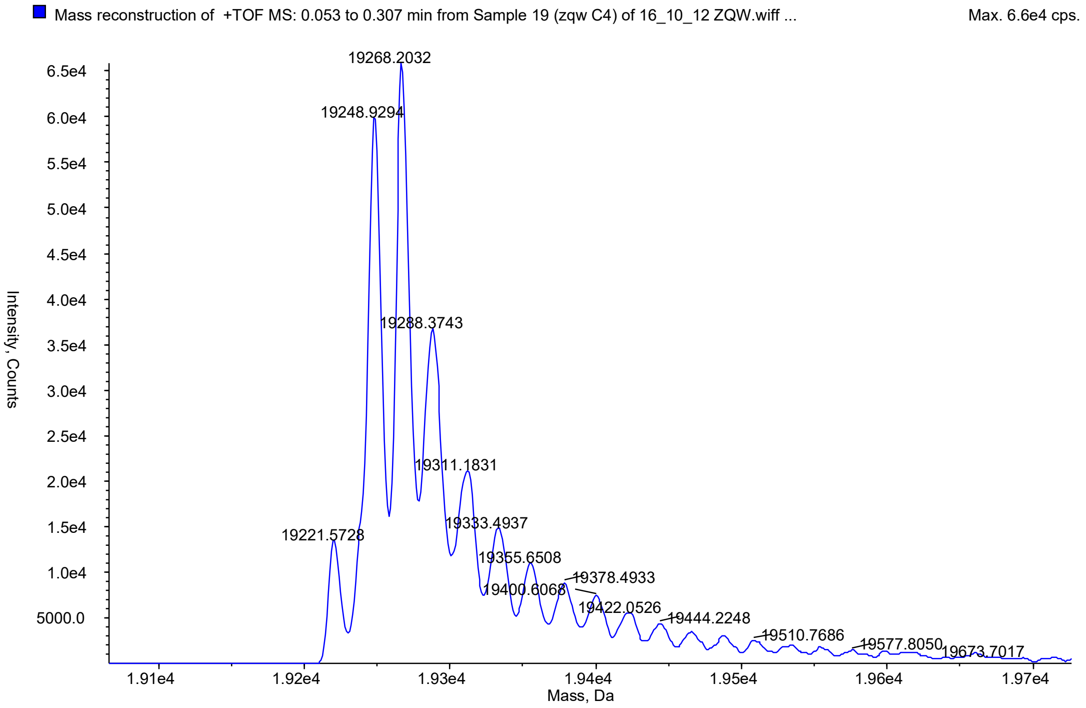

Figure S21. Mass spectral analysis of His$_6$-PpiB synthesized in the presence of 0.2 mM tyrosine and 2.0 mM *m*-fluorotyrosine (**15a**).

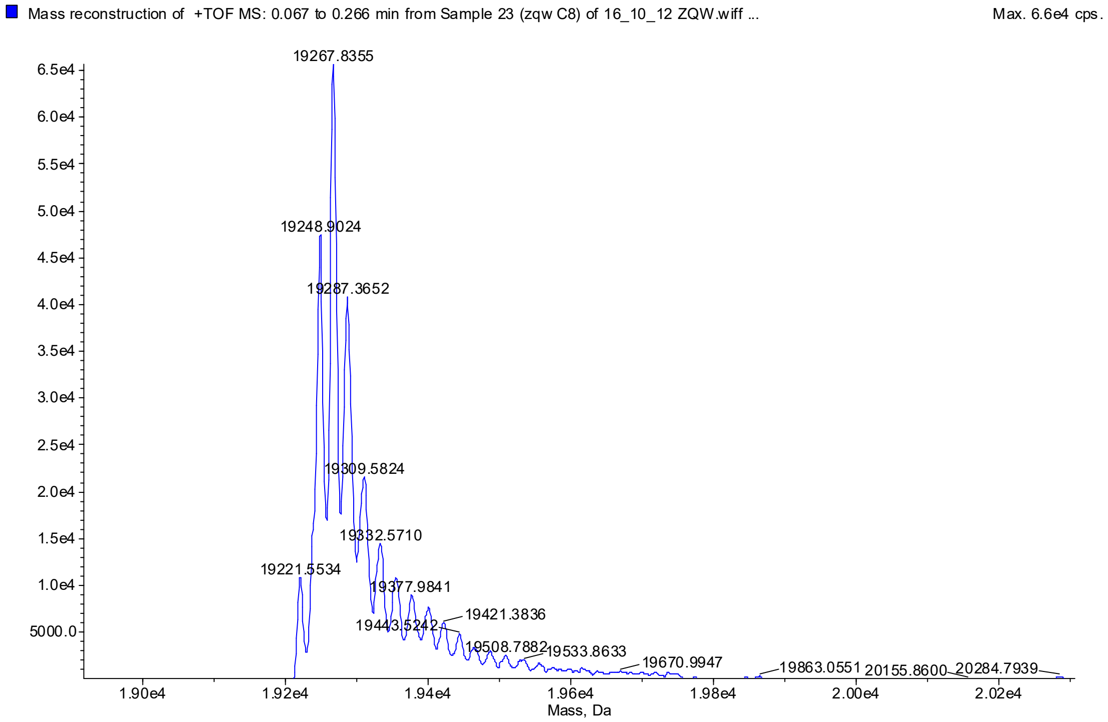

Figure S22. Mass spectral analysis of His$_6$-PpiB synthesized in the presence of 0.2 mM tyrosine and 2.0 mM *o*-fluorotyrosine (**15b**).

5. Representative HPLC of the aminoacylation of tyrosine

The K_M of Tyr with *E. coli* TyrRS has been reported to be 0.0061 mM. Therefore, a concentration of 0.2 mM, which is over 30 times greater than the K_M, was used in this assay. After pre-incubation of 0.2 mM Tyr with 0.05 μM *E. coli* His_6-TyrRS in tris buffer, in the presence of 1.0 mM ATP at 37 °C for one minute, reaction was initiated by adding *E. coli* $tRNA^{Tyr}$ to a total concentration of 60 μM. The reaction was allowed to proceed for 90 seconds. The rate of the aminoacylation reaction was determined by HPLC analysis of aliquots taken from the reaction mixture and quenched with 0.1% SDS every 15 seconds. At higher concentrations of *E. coli* His_6-TyrRS, aminoacylation occurs too fast to be monitored.

In a representative HPLC trace (Figure S23), peaks corresponding to AMP and ATP are observed at 8.1 min and 25.8 min, respectively. HPLC analysis of the reaction mixture shows that the level of AMP increases proportionally with time (Figure 5c). By comparison, the amount of AMP in the control stayed unchanged throughout the assay (Figure 5c). The low amount of AMP presenting in the control is presumably attributable to minor decomposition of ATP on storage. A separate HPLC analysis of ATP alone showed a comparable amount of AMP (Data not shown).

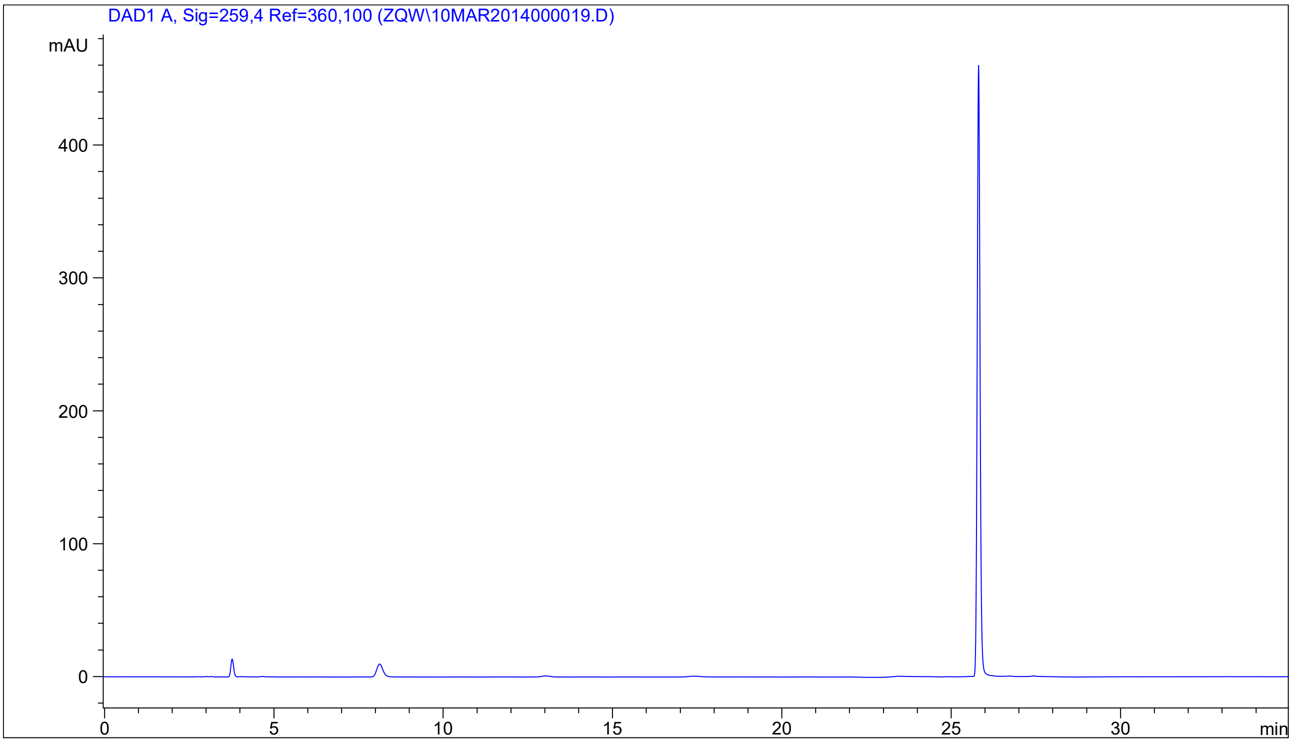

Figure S23. Representative HPLC trace. Analysis of aliquot taken from the reaction of Tyr after incubating for 90 seconds.

6. References

[1] A. Péter, E. Vékes, L. Gera, J. M. Stewart and D. W. Armstrong, A comparison of the direct and indirect LC methods for separating enantiomers of unusual glycine and alanine amino acid analogues. *Chromatographia*, 2002, **56**, S79-S89.

[2] D. Suarez, G. Laval, S. M. Tu, D. Jiang, C. L. Robinson, R. Scott and B. T. Golding, Benzylic brominations with *N*-bromosuccinimide in (trifluoromethyl) benzene. *Synthesis*, 2009, **2009**, 1807-1810.

[3] W. Q. Beard Jr, D. N. V. Eenam and C. R. Hauser, Ortho substitution rearrangement of certain 3-substituted and 3,5-disubstituted benzyltrimethylammonium ions by sodium amide1. *J. Org. Chem.,* 1961, **26**, 2310-2316.
[4] K. L. Cavanagh, S. A. Glover, H. L. Price and R. R. Schumacher, S_{N2} Substitution reactions at the amide nitrogen in the anomeric mutagens, *N*-acyloxy-*N*-alkoxyamides. *Aust. J. Chem.,* 2009, **62**, 700-710.
[5] A. S. Bailey, D. H. Bates, H. R. Ing and M. A. Warne, 2-3'5'-Dihydroxyphenylethylamine and 3-5-dihydroxy-phenylalanine. *J. Chem. Soc. Pak.,* 1952 (Nov), 4534-4535.
[6] T. Li, Y. Tsuda, K. Minoura, Y. In, T. Ishida, L. H. Lazarus, and Y. Okada, Enantioselective synthesis of a phenylalanine library containing alkyl groups on the aromatic moiety: confirmation of stereostructure by X-ray analysis. *Chem. Pharm. Bull.,* 2006, **54**, 873-877.
[7] H. Sugimoto, M. Ogata, H. Matsumoto, K. I. Sugita, A. Sato and T. Fujiwara, *U.S. Patent No. 5,326,780.* Washington, DC, 1994, U.S. Patent and Trademark Office.
[8] T. L. Sourkes, Enzymatic decarboxylation of 2, 6-and 2, 3-dihydroxyphenylalanine. *Can. J. Biochem. Physiol*., 1954, **32**, 515-518.
[9] G. J. M. Gruter, O. S. Akkerman and F. Bickelhaupt, Nuclear versus side-chain bromination of methyl-substituted anisoles by *N*-bromosuccinimide. *J. Org. Chem*., 1994, **59**, 4473-4481.
[10] D. Kuck, B. Paisdor and H. F. Grützmacher, Benzoanellierte centropolyquinane, 3 synthese mehrfach substituierter triptindane (9H, 10H-4b, 9a-([1,2] benzenomethano) indeno [1,2-a] indene) mit drei substituenten in ihrer Molekülhöhlung. *Chem. Ber.,* 1987, **120**, 589-595.
[11] A. Srikrishna and P. C. Ravikumar, The first total synthesis of (±)-γ-herbertenol, a herbertene isolated from a non-herbertus source. *Synthesis*, 2007, **2007**, 65-74.
[12] E. L. Bennett and C. Niemann, The synthesis of 2-fluoro-DL-tyrosine and 2-fluoro-4-methoxy-DL-phenylalanine. *J. Am. Chem. Soc.,* 1950, **72**, 1806-1807.
[13] T. J. McCord, D. R. Smith, D. W. Winters, J. F. Grimes, K. L. Hulme, L. Q. Robinson, L. D. Gage and A. L. Davis, Synthesis and microbiological activities of some monohalogenated analogs of tyrosine. *J. Med. Chem.,* 1975, **18**, 26-29.
[14] T. Nagasawa, T. Utagawa, J. Goto, C. J. Kim, Y. Tani, H. Kumagai and H. Yamada, Syntheses of L-tyrosine-related amino acids by tyrosine phenol-lyase of citrobacter intermedius. *Eur. J. Biochem*., 1981, **117**, 33-40.
[15] S. Khatib, O. Nerya, R. Musa, M. Shmuel, S. Tamir and J. Vaya, Chalcones as potent tyrosinase inhibitors: the importance of a 2, 4-substituted resorcinol moiety. *Bioorgan. Med. Chem*., 2005, **13**, 433-441.
[16] M. Hayashi, M. Shibuya and Y. Iwabuchi, Oxidation of alcohols to carbonyl compounds with diisopropyl azodicarboxylate catalyzed by nitroxyl radicals. *J. Org. Chem.,* 2012, **77**, 3005-3009.
[17] M. M. Khodaei, P. Ghanbary, I. Mohammadpoor-Baltork, H. R. Memarian, A. R. Khosropour and K. Nikoofar, Synthesis of 3-substituted indoles promoted by pulverization-activation method catalyzed by Bi (NO3) 3· $5H_2O$. *J. Heterocyclic. Chem*., 2008, **45**, 377-381.
[18] E. C. Jorgensen and R. A. Wiley, Methyl-substituted tyrosines and related compounds as potential anticancer agents. *J. Pharm. Sci*., 1963, **52**, 122-125.
[19] I. A. McDonald, P. L. Nyce, M. J. Jung and J. S. Sabol, Syntheses of DL-2-fluoromethy-p-tyrosine and DL-2-difluoromethyl-*p*-tyrosine as potential inhibitors of tyrosine hydroxylase. *Tetrahedron Lett*., 1991, **32**, 887-890.
[20] D. Suarez, G. Laval, S. M. Tu, D. Jiang, C. L. Robinson, R. Scott, and B. T. Golding, Benzylic brominations with N-bromosuccinimide in (trifluoromethyl) benzene. *Synthesis*, 2009, **2009**, 1807-1810.
[21] A. R. Katritzky, Y. J. Xu, A. V. Vakulenko, A. L. Wilcox and K. R. Bley, Model compounds of caged capsaicin: design, synthesis, and photoreactivity. *J. Org. Chem.,* 2003, **68**, 9100-9104.
